# Ultra–Efficient and Selective Gold Separation via Second–Sphere Coordination of Aurous Dihalide Using a Nonporous Amorphous Superadsorbent

**DOI:** 10.1002/advs.202501397

**Published:** 2025-03-05

**Authors:** Wei Zhou, Xiao Cai, Yiyao Xu, Min Zhou, Jialian Li, Qiang Liu, Qing He

**Affiliations:** ^1^ State Key Laboratory of Chemo/Biosensing and Chemometrics College of Chemistry and Chemical Engineering Hunan University Changsha 410082 P. R. China

**Keywords:** Covalent organic cage, Gold separation, Nonporous amorphous sorbent, Second‐sphere coordination, Superphane

## Abstract

The escalating demand for gold, coupled with dwindling terrestrial reserves, underscores the urgent need for innovative separation strategies, including e–waste recycling and seawater extraction. However, the development of ultra–efficient, highly selective adsorbents capable of recovering trace amounts of gold from complex aquatic matrices remains a formidable challenge. Herein, a covalent organic superphane cage is reported as a nonporous amorphous superadsorbent (NAS) for selective and efficient gold recovery via intermolecular second–sphere coordination of AuBr₂⁻ (or AuCl₂⁻) ions, subsequently converted to metallic gold through disproportionation. NAS demonstrates outstanding performance, including an exceptional gold uptake capacity of 2750 mg g⁻¹, ultrafast adsorption kinetics (40 s), broad pH tolerance (1–11, up to 6 m acids), and remarkable gold uptake even in 36 wt.% HCl solution (821 mg g⁻¹). NAS achieves over 99% selective gold recovery, even amidst excess competing ions, retaining efficacy across 30 regeneration cycles. Its versatile and scalable design enables applications in gold separation from gold‐bearing e–waste, catalytic residues, gold ores, and seawater. A large–scale trial recovered 23.8 Karat gold from printed circuit board leachates, positioning NAS as a sustainable and eco–friendly solution for industrial and environmental gold recovery.

## Graphical abstract

A covalent organic superphane cage serves as a nonporous amorphous superadsorbent for highly efficient and selective gold separation. This material uniquely captures linear aurous dihalide ions, achieving exceptional uptake capacity and selectivity, and proving effective even in seawater, offering a scalable, eco–friendly solution to gold scarcity challenges.

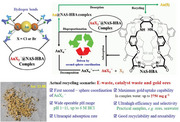

## 1 Introduction

Gold, one of the most esteemed precious metals, is renowned for its extraordinary electrical conductivity, superior ductility, chemical inertness, and unparalleled corrosion resistance.^[^ ^1^ ^]^ These exceptional properties have driven its extensive utilization across diverse sectors, including jewelry, medicine, and advanced electronics.^[^ ^1^, ^2^ ^]^ Consequently, the global demand for gold continues to escalate, posing significant challenges to its supply chain, particularly as traditional mining technologies rely predominantly on high–grade ores.^[^ ^3^ ^]^ To address this looming supply crisis, alternative strategies to augment global gold production are urgently required. These include the recovery of gold from electronic waste, extraction from lower–grade ores, and exploration of unconventional resources such as seawater.^[^ ^2^, ^4^ ^]^ Nonetheless, these gold–bearing sources pose substantial challenges: (1) the gold concentrations are extremely low, ranging from parts per million (ppm) to parts per trillion (ppt); (2) complex matrices of interfering organic and inorganic species hinder selective extraction; and (3) competing components often exist in significantly higher concentrations. Current gold extraction technologies are further constrained by complex processes, high energy consumption, and severe environmental repercussions from hazardous waste emissions.^[^ ^3^, ^5^ ^]^ In light of these challenges, there is an urgent need for innovative, sustainable approaches to gold separation that minimize environmental impact while maximizing efficiency. Advanced adsorbents are poised to play a transformative role in addressing these pressing issues, offering the potential to selectively capture trace amounts of gold from challenging matrices and contribute to a sustainable supply chain.

Gold exhibits a diverse range of oxidation states from –I to +V, but in aqueous solutions, it predominantly exists as Au(I) and Au(III), forming linear two–coordinate and square planar complexes, respectively.^[^ ^6^ ^]^ Among these, cyanide and halide complexes are the most significant and widely encountered. In particular, the cyanide complex [Au(CN)₂]⁻ plays a critical role in gold leaching from ores and e–wastes, underpinning the extensive use of cyanide in hydrometallurgical processes.^[^ ^7^ ^]^ However, these processes generate hazardous cyanide–laden waste, contribute to substantial carbon emissions, and require high energy inputs, raising pressing environmental and safety concerns.^[^ ^8^ ^]^

Efforts to address these issues have led to the development of materials such as cyclodextrins, bambusuril macrocycles, and chitosan fibers for binding and adsorbing dicyanoaurate.^[^ ^7^, ^9^ ^]^ Despite these advancements, there is an urgent need for materials that target cyanide–free gold complexes to mitigate ecological and safety risks. In this context, halide complexes, particularly chlorides and bromides, present compelling alternatives due to their environmental compatibility and prevalence in nature and industry.^[^ ^10^ ^]^ Gold chloride/bromide complexes are found in *aqua regia*–leached liquors from ores and e–wastes, seawater, and gold–containing industrial wastewater, making them ideal targets for sustainable gold recovery strategies.^[^ ^5^, ^11^ ^]^

**Figure 1:**
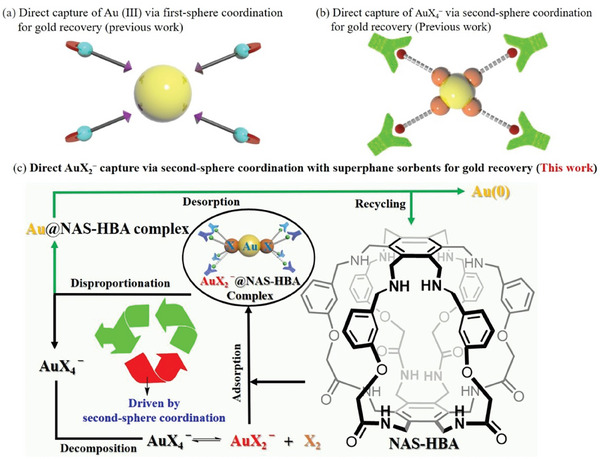
Working mechanisms for gold separation. a) Direct capture of Au(III) via first–sphere coordination as the driving force (known); b) direct capture of AuX_4_^−^ via second–sphere coordination (known); c) direct AuX_2_^−^ capture via second–sphere coordination and the gold recovery profile reported in this work. The energetically favorable AuX_2_^−^ binding through second–sphere coordination offsets the endergonic nature of the decomposition reaction AuX_4_^−^ ⇌ AuX_2_^−^ + X_2_, effectively propelling this reaction forward.

A wide array of materials, including porous carbon, functional resins, modified cellulose, ionic liquids, metal–organic frameworks (MOFs), covalent organic frameworks (COFs), and supramolecular macrocycles, has been developed for gold separation via extraction, adsorption, or precipitation.^[^ ^12^ ^]^ Most of these systems operate by directly coordinating Au(III) with ligands, followed by chemical or electrochemical reduction to release Au(0), facilitating the recycling of sorbents or extractants (**Figure** **1**a).^[^ ^12^, ^13^ ^]^ However, systems capable of capturing AuX_4_^−^ (X = Cl or Br) via second–sphere coordination remain rare (**Figure** 1b).^[^ ^13^, ^14^ ^]^ Notably, AuX_2_^−^ species, commonly present in seawater and formed from the decomposition of AuX_4_^−^ (AuX_4_^−^ ⇌ AuX_2_^−^ + X₂), offer a largely untapped opportunity for gold recovery. To the best of our knowledge, the development of sorbents specifically designed to selectively target AuX_2_^−^ through second–sphere coordination remains uncharted. This innovation holds the potential to revolutionize gold recovery from low–grade ores, e–wastes, and seawater, enabling unprecedented levels of selectivity and efficiency.

Adsorption processes are often preferred for metal, especially gold, separation due to their operational simplicity, environmental compatibility, and cost efficiency. Consequently, the development of advanced adsorption materials for gold recovery has emerged as a critical area of research over the past decades. Typically, state–of–the–art gold adsorbents require porous materials, such as MOFs and COFs, with high porosity or large surface areas to achieve superior adsorption capacity and rapid kinetics.^[^ ^13^, ^15^ ^]^ In this study, we report a groundbreaking discovery that a discrete, purely covalent organic superphane cage (**NAS–HBA**, **Figure** 1c) can function as a nonporous amorphous superadsorbent (**NAS**) with exceptional performance for sustainable gold separation. Single–crystal structure analysis of the **NAS–HBA**–Aurous bromide complex reveals external binding pockets that capture linear AuBr₂⁻ species, while bromide dihydrate is stabilized inside the internal cavity of **NAS–HBA**. Remarkably, each AuBr₂⁻ unit coordinates with four **NAS–HBA** cages, and each cage interacts with four AuX_2_^−^ units, forming unprecedented AuBr₂⁻–mediated supramolecular organic frameworks (SOFs). Contrary to the reliance on porosity observed in conventional gold adsorbents, the as–prepared nonporous amorphous cage powders achieve an extraordinary gold uptake capacity of up to 2750 mg g⁻¹, even in strongly acidic environments (pH 1–11, and up to 6 M acids) with high levels of interfering ionic species. AuX_2_^−^ species captured by **NAS–HBA** rapidly undergo disproportionation to yield Au(0) and AuX_4_^−^, as confirmed by comprehensive analytical techniques including powder X–ray diffraction (PXRD), Fourier transform infrared (FTIR), Raman spectroscopy, X–ray photoelectron spectroscopy (XPS), and Ultraviolet–visible (UV–Vis) spectroscopy. **NAS–HBA** demonstrates exceptional efficiency and selectivity in extracting trace gold (from ppm to ppb levels) from *aqua regia* leachates of electronic waste, such as discarded computer processing units (CPUs) and printed circuit boards (PCBs), as well as from catalytic waste and mining ores, achieving separation efficiencies of up to 99.5%. The gold recovered from e–waste exhibits a purity exceeding 99% (equivalent to 23.8 Karat). Furthermore, ultratrace gold concentrations (as low as 10 ppb) in the Xiangjiang River or seawater can be effectively removed using **NAS–HBA**, which retains excellent recyclability and reusability, highlighting its potential for practical and sustainable applications.

## 2 Results and Discussion

### 2.1 Single–Crystal Structure of the AuBr_2_^−^ and **NAS–HBA** Complex

In complex and dynamic environments, nature often provides the most sophisticated solutions. For example, marine organisms demonstrate remarkable efficiency in enriching essential metal ions from seawater to sustain their physiological functions. This ability relies on specialized transporters or proteins designed for selective metal absorption and transport, for example, SLC39A as Zn transporters. Inspired by these natural processes, we have sought to engineer artificial adsorbents mimicking such protein structures to achieve highly efficient and selective metal ion adsorption. In this pursuit, we independently established a novel class of covalent organic superphane cages, distinguished by their unique architecture of two face–to–face benzene rings connected by six bridges.^[^ ^16^ ^]^ These nearly enclosed structures, densely populated with recognition sites, exhibit exceptional stability for labile water clusters and ion hydrates and have shown promise in the selective adsorption of iodine species (I₂/I₃⁻).^[^ ^16b,c^ ^]^ Notably, superphane **NAS–HBA** has demonstrated the capacity to encapsulate ReO₄⁻ ions within its internal cavity, efficiently and selectively extracting perrhenate from waste simulants.^[^ ^16a^ ^]^

**Figure 2:**
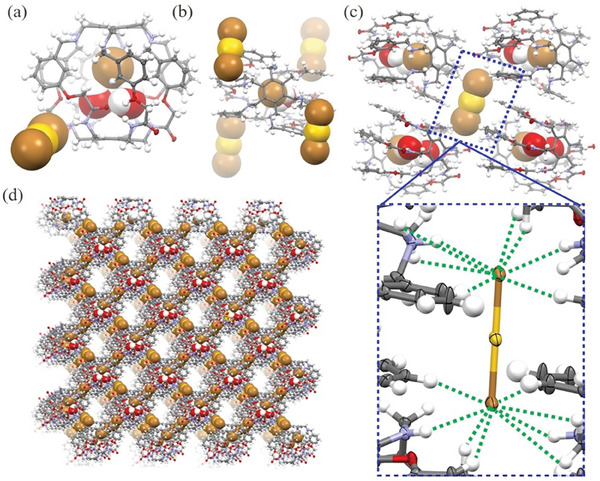
Single–crystal structure of Br^−^·2H_2_O@**NAS–HBA**·2H^+^·AuBr_2_^−^ complex. a) a snapshot of the complex with bromide dihydrate and AuBr_2_^−^ shown in space–filling model; b) one selected Br^−^·2H_2_O@**NAS–HBA**·2H^+^ complex surrounded by four AuBr_2_^−^ ions; c) one selected AuBr_2_^–^ interacting with four Br^−^·2H_2_O@**NAS–HBA**·2H^+^ complexes through multiple hydrogen bonds as indicated by green dashed lines (short C/N─H···Br contacts of 2.72 – 3.46 Å); d) the AuBr_2_^−^–mediated 3D supramolecular organic frameworks.

Building upon these findings, we hypothesized that the six secondary amine and six amide units of **NAS–HBA**, combined with its internal binding cavity and external complexation environment, could enable unique interactions with gold halide complexes. **NAS–HBA** was synthesized and comprehensively characterized following our previously reported method.^[^ ^16a^ ^]^ When **NAS–HBA** was employed in a chloroform–based solid–liquid extraction of NaAuBr₄, single–crystal analysis revealed an unexpected structure. Instead of encapsulating AuBr₄⁻ within its internal cavity, the crystal structure displayed a Br⁻·2H₂O@**NAS–HBA**·2H+ complex, with a linear AuBr₂⁻ molecule bound externally to the cage (**Figure** **2**a; Figure S1, Supporting Information). The internal cavity was fully occupied by a Br⁻·2H₂O complex. Interestingly, each Br⁻·2H₂O@**NAS–HBA**·2H+ complex directly interacted with four linear AuBr₂⁻ units (**Figure** 2b; Figure S2, Supporting Information), while each AuBr₂⁻ engaged four Br⁻·2H₂O@**NAS–HBA**·2H+ complexes via up to 16 hydrogen bonds (C─H···Br and N─H···Br), as indicated by green dashed lines in **Figure** 2c and Figure S2 (Supporting Information). These H···Br distances, ranging from 2.72 Å to 3.46 Å, indicate moderate to weak hydrogen bonding interactions, enabling the external stabilization of AuBr₂⁻ through second–sphere coordination. These interactions culminated in the formation of a remarkable 3D AuBr₂⁻–mediated supramolecular organic framework (**Figure** 2d; Figures S3 and S4, Supporting Information). Unlike the more stable planar AuBr₄⁻ species, the linear AuBr₂⁻ complex is inherently less stable, making its successful capture within a lattice structure particularly notable. To the best of our knowledge, this represents one of the rare instances where dibromo–gold(I) has been stabilized and organized into a crystalline lattice.^[^ ^17^ ^]^

### 2.2 Direct AuBr_2_^−^ Adsorption by Nonporous Amorphous **NAS–HBA**

**Figure 3:**
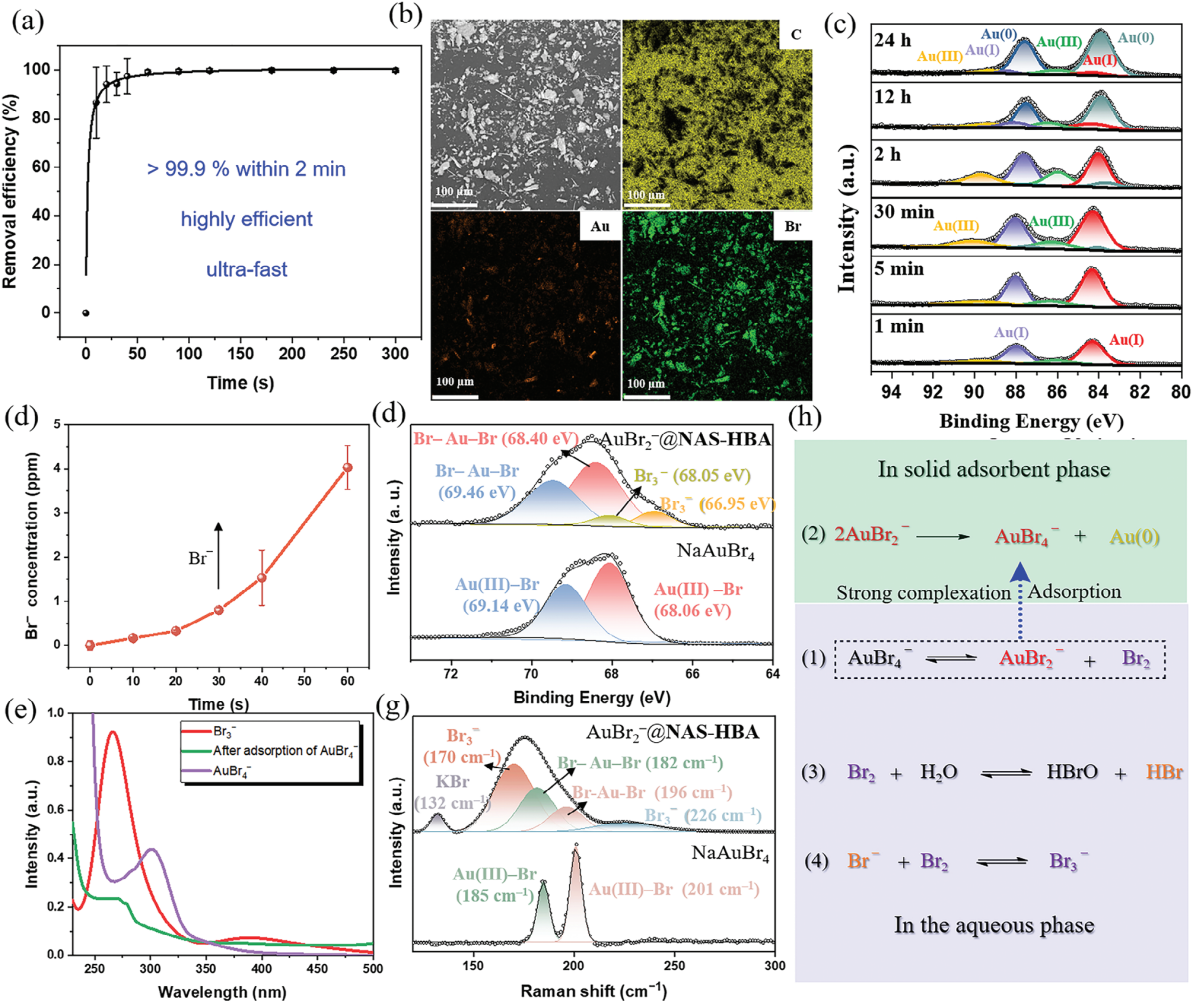
Direct AuBr_2_^−^ adsorption by **NAS–HBA**. a) Time–resolved gold recovery efficacy. b) Post–adsorption **NAS–HBA** morphology via SEM and elemental composition via EDS, c) Temporal high–resolution XPS tracking of gold species evolution. d) Bromide ion concentration assay during adsorption. e) Comparative UV–vis spectral analysis of AuBr_4_^−^ (0.01 mm), Br_3_^−^ (0.1 mm) and post–**NAS–HBA** treatment of AuBr_4_^−^ (4 mm) in water. f) Br *3d* XPS characterization pre– and post–adsorption, g) Raman spectroscopic assessment of NaAuBr_4_ and **NAS–HBA** after gold adsorption. h) The hypothesized mechanism and evidences for direct adsorption of AuBr_2_^−^. Data represent mean ± SD from n = 3 independent experiments.

The unexpected discovery of AuBr₂⁻ capture (arising from the equilibrium reaction AuBr₄⁻ ⇌ AuBr₂⁻ + Br₂) within the single–crystal structure of **NAS–HBA** prompted us to investigate its potential for gold adsorption from aqueous solutions. To test this hypothesis, we performed adsorption experiments using the as‐synthesized **NAS–HBA**, a discrete covalent organic cage confirmed to be nonporous and amorphous (Figure S5, Supporting Information). In a representative experiment, 4.0 mg of **NAS–HBA** was immersed in 4 mL of a NaAuBr₄ aqueous solution (20 ppm) and agitated with a magnetic stirrer. The residual gold concentration in the solution was monitored by inductively coupled plasma mass spectrometry (ICP–MS), revealing an impressive gold removal efficiency of 99.9% within just 2 min (**Figure** **3**a). This demonstrates the exceptional efficacy and rapid adsorption kinetics of **NAS–HBA** as a nonporous amorphous sorbent (**NAS**).

Following adsorption, the **NAS–HBA** material was filtered and analyzed by scanning electron microscopy (SEM) coupled with energy–dispersive X–ray spectroscopy (EDS), confirming the presence of C, N, O, Au, and Br elements and verifying successful gold uptake (**Figure** 3b; Figure S6, Supporting Information). XPS was employed to characterize the adsorbed gold species over time (**Figure** 3c). Initially, during the first 1 to 30 min of adsorption, Au(I) was the predominant species on **NAS–HBA**, with traces of Au(III). As the adsorption time extended to 2 h, the relative amount of Au(III) increased transiently before declining, reflecting a dynamic equilibrium. Remarkably, peaks corresponding to Au(0) began to emerge after 12 h, becoming dominant by 24 h, while peaks for Au(I) diminished significantly. This transformation was corroborated by PXRD, which showed characteristic diffraction peaks of metallic gold (Au(0), Figure S7, Supporting Information).

Fourier transform infrared (FTIR) spectroscopy further revealed that the **NAS–HBA** framework remained largely unchanged after 24 h of gold adsorption (Figure S8, Supporting Information). Importantly, these results suggest that the formation of metallic gold did not arise from the direct reduction of Au(I) or Au(III) at the expense of sorbent oxidation, a mechanism often observed in similar systems.^[^ ^13g,j,k^ ^]^ Instead, we propose that the conversion of Au(I) to Au(0) follows an energetically favorable disproportionation reaction: 3AuBr₂⁻ ⇌ AuBr₄⁻ + 2Au(0) + 2Br⁻ (reaction Equation 4).^[^ ^19^ ^]^ This unique mechanism, which preserves the structural integrity of **NAS–HBA**, represents a significant departure from conventional pathways, highlighting the innovative potential of this system for sustainable gold separation.^[^ ^13^, ^15^, ^20^ ^]^

Next, we aimed to investigate the chemical processes occurring in the solution phase during gold adsorption. Notably, ionic chromatography revealed a sharp increase in bromide concentration within the first minute of adsorption, indicating its release during the reaction (**Figure** 3d). Furthermore, UV–vis spectroscopy identified a distinct absorbance peak corresponding to Br₃⁻ after gold adsorption from an AuBr₄⁻ solution by **NAS–HBA** powder (**Figure** 3e). Complementary XPS analysis of the **NAS–HBA** solid post–adsorption revealed two characteristic peaks for Br₃⁻ at 66.95 and 68.05 eV, alongside signals attributable to AuBr₂⁻ at 68.40 and 69.46 eV (**Figure** 3f).^[^ ^21^ ^]^ In contrast, pristine NaAuBr₄ exhibited Br *3d* peaks at 68.06 and 69.14 eV, consistent with its expected electronic configuration.

Raman spectroscopy further corroborated these findings, showing peaks at 185 and 201 cm^−1^ associated with Au(III)–Br stretching vibrations in solid NaAuBr₄. After adsorption, the Raman spectrum of **NAS–HBA** revealed new peaks indicative of Br₃⁻ (170 and 226 cm^−1^) and AuBr₂⁻ (182 and 196 cm^−1^) (**Figure** 3g).^[^ ^21^, ^22^ ^]^ These results collectively suggest that during NaAuBr₄ adsorption in aqueous solutions, AuBr₄⁻ undergoes partial decomposition via an endergonic equilibrium reaction AuBr₄⁻ ⇌ AuBr₂⁻ + Br₂ (equiation 1), yielding trace amounts of AuBr₂⁻ and Br₂ (Figure 3h).

The evolution of the N *1s* XPS spectrum of **NAS–HBA** during AuBr₄⁻ adsorption provides further insight into the adsorption mechanism. After 1 min of adsorption, two distinct peaks at 401.26 eV and 399.40 eV, corresponding to C–NH₂+ and C–NH, respectively, were observed.^[^ ^16b^ ^]^ These peaks exhibited slight shifts to 401.32 and 399.42 eV after 24 h of adsorption (Figure S9, Supporting Information). In comparison, the N *1s* spectrum of fresh **NAS–HBA** displayed only a single peak at 398.87 eV. This suggests that **NAS–HBA** undergoes protonation in acidic aqueous environments, forming C–NH₂+ and C–NH, which subsequently engage in hydrogen bonding and electrostatic interactions with AuBr₂⁻ species in solution.^[^ ^13^, ^14^ ^]^ The O *1s* XPS spectra also revealed minor shifts following AuBr₄⁻ adsorption. For fresh **NAS–HBA**, peaks were observed at 532.51 and 530.75 eV, corresponding to C═O and C─O groups, respectively. After 24 h of adsorption, these peaks shifted slightly to 532.38 and 530.67 eV (Figure S10, Supporting Information). This subtle shift suggests that the interaction between oxygen–containing groups and Au(III) or Au(I) does not involve strong first–sphere coordination, in agreement with previous studies.^[^ ^13^, ^15^, ^23^ ^]^ Instead, the observed decrease in binding energy can be attributed to weaker interactions of C═O and C─O groups with Au(0), consistent with earlier findings.^[^ ^13^, ^24^ ^]^

The highly acidic conditions significantly suppress the potential for direct coordination between Au(I) and the nitrogen or oxygen atoms of the **NAS–HBA** framework.^[^ ^13^, ^20^, ^25^ ^]^ In aqueous solutions, AuBr₄⁻ remains a stable gold species, with only trace amounts of AuBr₂⁻ generated through the unfavorable equilibrium AuBr₄⁻ ⇌ AuBr₂⁻ + Br₂ (Equation 1). The Br₂ produced can subsequently transform into HBr and HBrO through the equilibrium H₂O + Br₂ ⇌ HBr + HBrO (Equation 2). Under standard conditions, AuBr₂⁻ and Br₂ are nearly undetectable, and the solution pH remains unchanged (Table S1, Supporting Information). However, in the presence of **NAS–HBA**, the pH of the gold–containing solution after adsorption decreased significantly, from 4.84 to 2.31. This observation can be rationalized by the favorable binding of **NAS–HBA** to linear AuBr₂⁻, effectively driving the unfavorable equilibrium AuBr₄⁻ ⇌ AuBr₂⁻ + Br₂ forward. The resulting Br₂ reacts with water to form HBr and HBrO, causing the observed decrease in pH. Although the binding of AuBr₂⁻ within the internal cavity of **NAS–HBA** cannot be entirely excluded, the cooperative hydrogen bonding and electrostatic interactions indicated by the single–crystal structure strongly could support major external binding.

The minor Br⁻ generated in this reaction can be captured by Br₂, forming Br₃⁻ via the equilibrium Br⁻ + Br₂ ⇌ Br₃⁻ (Equation 3), as experimentally verified (**Figure** 3e,g). Thus, the complexation of AuBr₂⁻ appears to be the driving force behind this unique aurous dibromide adsorption. At pH 1, UV–vis spectra revealed characteristic peaks at 318 nm and 228 nm, attributed to the formation of NaAuCl₄ due to the exchange of Cl⁻ from HCl (Figure S11a, Supporting Information) ^[^ ^6^, ^26^ ^]^. After 24 h, the peak intensities remained largely unchanged, indicating negligible decomposition or disproportionation of AuCl₄⁻, confirming its stability at acidic pH. Similarly, at neutral pH, NaAuBr₄ exhibited no significant interference, with its characteristic peaks remaining stable over 24 h (Figure S11b, Supporting Information).^[^ ^6^, ^26^ ^]^ However, at pH 14, the Br⁻ ligands in NaAuBr₄ were rapidly replaced by OH⁻, forming AuOH₄⁻ (Figure S11c, Supporting Information).^[^ ^26^, ^27^ ^]^ This stable species likely accounts for the reduced adsorption efficiency observed under alkaline conditions (Figure S12, Supporting Information).

In the solid state, the adsorbed AuBr₂⁻ undergoes a disproportionation reaction, resulting in its transformation into AuBr₄⁻ and metallic gold (Au(0)). PXRD analysis revealed that after 30 min of AuBr₄⁻ adsorption, **NAS–HBA** transitioned from an amorphous to a crystalline state, whereas control samples not exposed to AuBr₄⁻ exhibited no such phase change. Interestingly, the PXRD spectra of **NAS–HBA** post–adsorption did not perfectly align with the simulated single–crystal spectra. This discrepancy is hypothesized to arise from the dynamic nature of the adsorption process, which likely induces structural changes that are not fully captured in the static single–crystal model (Figure S13, Supporting Information).

Comparable results were observed for AuCl₄⁻, irrespective of the associated cations (Na+, K+, or NH₄+), underscoring the versatility of **NAS–HBA** in gold halide complex adsorption (Figures S14–S31, Supporting Information). These findings support the conclusion that superphane–based **NAS–HBA** solids can directly capture AuX_2_^−^ species, subsequently converting them into metallic gold via a disproportionation reaction. To the best of our knowledge, this represents the first reported instance of gold recovery achieved through the direct and efficient capture of linear, metastable AuX_2_^−^ ions, marking a significant departure from conventional approaches that primarily target Au^3^+ and AuX_4_^−^ ions. This novel mechanism highlights the potential of **NAS–HBA** as a transformative material for advanced gold recovery applications.

### 2.3 Gold Adsorption Kinetics, Maximum Uptake Capacity, pH Tolerance, Selectivity and Recyclability

**Figure 4:**
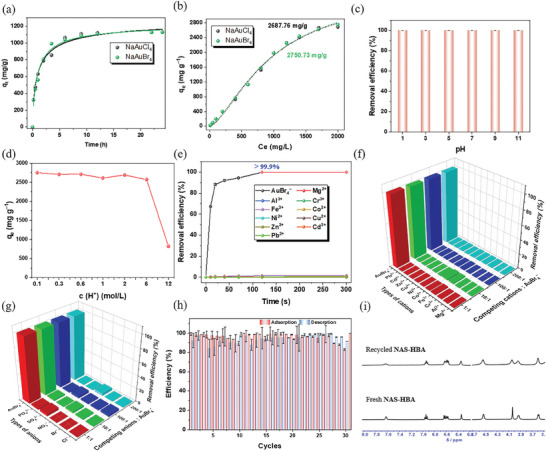
Gold adsorption performance of **NAS–HBA**. a) The adsorption kinetics of AuBr_4_^–^ (in green) and AuCl_4_^–^ (in black) solutions with the concentration of 600 ppm. b) The dependence of gold adsorption capacity on adsorption concentration, Ce is the concentration of Au(III) in aqueous solution before adsorption. c) The effect of pH value on the adsorption of AuBr_4_^–^ over **NAS–HBA**. d) The dependence of gold adsorption capacity on concentration of hydrochloric acid. e) The adsorption rate of gold from complex liquids containing 20 ppm of NaAuBr_4_ in the presence of 1 equivalent of equal–mass Mg^2+^, Al^3+^, Cr^3+^, Fe^3+^, Co^2+^, Ni^2+^, Cu^2+^, Zn^2+^, Cd^2+^ and Pb^2+^ (as their chloride salts) using **NAS–HBA** solid. f) Removal efficiency (relative uptake) of ions from aqueous AuBr_4_^–^ (20 ppm) solutions containing an excess (1 to 200 folds) of equal–mass competing cations, viz. Mg^2+^, Al^3+^, Cr^3+^, Fe^3+^, Co^2+^, Ni^2+^, Cu^2+^, Zn^2+^, Cd^2+^ and Pb^2+^. g) Removal efficiency (relative uptake) of ions from aqueous AuBr_4_^–^ (20 ppm) solutions containing an excess (1 to 200 folds) of equal–mass competing cations, viz. Cl^−^, Br^−^, NO_3_^−^, SO_4_^2−^ and PO_4_^3−^. h) The gold removal efficiency with recycled adsorbent **NAS–HBA** during 30 cycles in the batch adsorption experiments. i) Partial ^1^H NMR spectra of fresh (bottom) and recycled (top) **NAS–HBA** in DMSO–*d_6_* after undergoing 30 adsorption–desorption cycles. Error bars represent SD. n = 3 independent experiments.

As demonstrated in Figure 3a, the nonporous amorphous superadsorbent (**NAS–HBA**) rapidly and nearly completely removed gold from a 20 ppm AuBr_4_^−^ aqueous solution within 60 s, underscoring its exceptionally fast gold adsorption kinetics. When exposed to high concentrations of AuBr_4_^−^ or AuCl_4_^−^ (600 ppm) in water, an initial rapid adsorption phase was observed, followed by a gradual increase over 24 h at a slower rate (**Figure** **4**a; Figure S32, Supporting Information). This deceleration and the gradual approach to saturation can be attributed to the rapid initial binding of AuBr_2_^−^ or AuCl_2_^−^ ions outside the sorbents' cavities, succeeded by a disproportionation reaction that converts the adsorbed gold species into metallic gold. This transformation not only generates additional adsorption sites within the superadsorbent but also facilitates continuous gold uptake until the ion source is exhausted. The adsorption capacity of **NAS–HBA** increases significantly with the initial concentration of gold ions, ranging from 10 ppm to 2000 ppm, and peaks at approximately 2687 mg g^−1^ for AuCl_4_^−^ and 2750 mg g^−1^ for AuBr_4_^−^ (**Figure** 4b; Figure S33, Supporting Information). These remarkable values position **NAS–HBA** as one of the most effective materials for gold recovery, rivaling state–of–the–art porous systems such as MOFs, COFs, and porous organic polymers (POPs) (Table S2, Supporting Information).

To further evaluate the versatility of **NAS–HBA**, we investigated its pH tolerance, a critical parameter for sorbent materials in real–world applications. Remarkably, **NAS–HBA** demonstrated outstanding gold separation efficiency, achieving near–complete removal (≈100%) across a wide pH range from 1 to 11 (**Figure** 4c; Figure S34, Supporting Information). This exceptional pH adaptability underscores **NAS–HBA**’s potential as a robust and versatile sorbent for gold recovery in diverse environmental conditions, ranging from acidic to basic solutions. Notably, **NAS–HBA** maintained an impressive gold uptake capacity of ≈2700 mg g⁻¹, even in highly acidic environments, such as 6 mol L^−1^ HCl. In extremely acidic conditions, including 36 wt.% HCl aqueous solutions, **NAS–HBA** exhibited a remarkable uptake capacity of 821 mg g⁻¹, representing one of the few reported materials capable of such high–efficiency Au(III) adsorption in concentrated hydrochloric acid (**Figure** 4d; Table S2, Supporting Information).^[^ ^13^, ^15^, ^25^, ^28^ ^]^ This chemical resilience was further validated through partial ¹H NMR spectroscopy of **NAS–HBA** after 24 h of stirring in hydrochloric acid solutions of varying concentrations (pH 3, 1 m, 2 m, 6 m, and 36 wt.% HCl), which revealed the presence of six protons or charges associated with the C–NH–C units. These results confirm **NAS–HBA**'s remarkable stability and robust performance under extreme acidic conditions (Figures S35 and S36, Supporting Information).

To evaluate the selectivity of **NAS–HBA** for gold adsorption, we systematically examined its performance against various competing ions, including Mg^2^^+^, Al^3^^+^, Cr^3^^+^, Fe^3^^+^, Co^2^^+^, Ni^2^^+^, Cu^2^^+^, Zn^2^^+^, Cd^2^^+^, and Pb^2^^+^, using simulated gold ore solutions containing either AuBr₄⁻ or AuCl₄⁻ (as Na^+^ or K^+^ salts). At neutral pH (pH 7), **NAS–HBA** maintained high gold removal efficiency (>89%), although certain cations, such as Cr^3^^+^ (31%), Cu^2^^+^ (30%), and Pb^2^^+^ (34%), were also adsorbed from a gold–containing solution (20 ppm) in the presence of equimolar concentrations of competing ions (Figure S37, Supporting Information). However, upon reducing the pH to 3, the adsorption of these competing cations was significantly diminished, and at pH 1, **NAS–HBA** achieved 99.9% gold removal within just 120 s, while the removal of competing cations remained negligible even after 300 s (**Figure** 4e; Figure S38 and S39, Supporting Information). The selectivity of **NAS–HBA** is further highlighted by its distribution coefficient (*K_d_*) values, which exceeded 10^6^ mL g⁻¹ for gold, compared to *K_d_* values below 39 mL g⁻¹ for competing cations (Figure S40, Supporting Information). The calculated separation coefficients (*α*, defined as K_Au_/K_cations_) underscore this selectivity, with values surpassing 10^5^ for gold compared to other cations (Figure S41, Supporting Information). Additional adsorption tests using **NAS–HBA** (4 mg) with 20 ppm solutions of Au (as NaAuCl₄), Pd (as Na₂PdCl₄), Pt (as Na₂PtCl₄), and Ag (as AgNO₃) at pH 1 revealed the following selectivity trend: Au (99.9%) >> Pd (44.6%) > Pt (8.3%) > Ag (3.1%) (Figures S42 and S43, Supporting Information). This exceptional selectivity is attributed to the highly acidic environment, which suppresses coordination interactions between competing metal ions and the heteroatoms (O and N) of the sorbent.

Moreover, **NAS–HBA** maintained its remarkable selectivity for gold in the presence of 200–fold excess competing cations (Mg^2^+, Al^3^+, Cr^3^+, Fe^3^+, Co^2^+, Ni^2^+, Cu^2^+, Zn^2^+, Cd^2^+, and Pb^2^+) or a 200–fold excess of common anions (Cl⁻, Br⁻, NO₃⁻, SO₄^2^⁻, and PO₄^3^⁻) in 2 M acidic solutions (**Figure** 4f; Figure S44, Supporting Information). These prevalent inorganic anions, often found in gold–containing sources, showed minimal interference with the gold adsorption process, underscoring **NAS–HBA**’s unparalleled efficiency and selectivity for gold recovery under challenging conditions. To assess the impact of competing anions on gold adsorption by **NAS–HBA**, we introduced 4.0 mg of **NAS–HBA** to a gold solution (AuBr₄⁻ or AuCl₄⁻ at 20 ppm) in water (4 mL), supplemented with equimolar masses of Cl⁻, Br⁻, NO₃⁻, SO₄^2^⁻, and PO₄^3^⁻ (as their Na+ salts). Remarkably, **NAS–HBA** demonstrated near–complete gold adsorption with negligible adsorption of competing anions (**Figure** 4g; Figures S45–S48, Supporting Information). The distribution coefficients (*K_d_*) highlight the material's exceptional selectivity for Au(III), with values exceeding 10⁷ mL g⁻¹, compared to *K_d_* values below 77 mL g⁻¹ for other anions (Figure S49, Supporting Information). Separation coefficients further emphasize this superiority, with values greater than 10^6^ for gold in the presence of competing anions (Figure S50, Supporting Information). Even under extreme conditions–such as the presence of a 200–fold excess of competing anions in 2 M acidic solutions or a 1000:1000:1 mass ratio of Cl⁻ and NO₃⁻ to gold–**NAS–HBA** maintained its gold adsorption efficiency, underscoring its robustness (Figure S51, Supporting Information). The adsorption process adhered to the Langmuir pseudo–second–order kinetic model, with a high correlation coefficient (R^2^ > 0.99), confirming the model's applicability and providing a clear understanding of the adsorption mechanism (Figures S15,  S32b,  S39, and S47, Supporting Information).

Recyclability and reusability are critical for the sustainable and cost–effective application of sorbents. While traditional systems often utilize thiourea and HCl for elution, we opted for a thiourea and K₂CO₃ solution to release metallic gold and regenerate **NAS–HBA** for reuse. Specifically, 300 mg of **NAS–HBA** was employed to adsorb gold from an acidic AuBr₄⁻ solution (100 ppm, pH 1) within 2 min. The sorbent was then treated with a 5 wt.% thiourea and 0.25 M K₂CO₃ solution at 40 °C for 4 h, followed by filtration, drying, and reuse. Notably, the gold recovery efficiency of the regenerated **NAS–HBA** matched that of the virgin material, even after 30 cycles (**Figure** 4h). This exceptional performance underscores **NAS–HBA**'s outstanding recyclability and reusability. Structural integrity was confirmed through ¹H NMR and FT–IR analyses, which revealed no significant changes in **NAS–HBA** after 30 cycles of adsorption–desorption (**Figure** 4I; Figure S52, Supporting Information). These results firmly establish the robustness and efficiency of **NAS–HBA**, making it a promising candidate for practical gold extraction from diverse and complex matrices, including e–waste leachates, gold ores, and industrial waste streams.

### 2.4 Practical Separation of Gold from Catalytic Residues, e–Wastes, Ores, and Seawater

**Figure 5:**
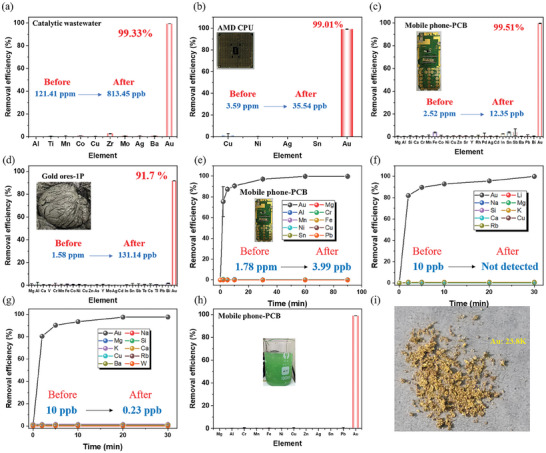
The gold separation performance of **NAS–HBA** from real–world samples. a) Gold–containing catalytic wastewater; *aqua regia* leaching solutions obtained from b) the AMD CPUs, c) the mobile phone of PCBs, d) the gold ores; e) wastewater from Xiangjiang River (C_0_ = 10 ppb); f) seawater from The Yellow Sea (C_0_ = 10 ppb); g) the NBS/Py leaching solutions of mobile phone PCBs; h) the scaled–up selective gold adsorption from the NBS/Py leaching solutions of mobile phone PCBs; and i) the image of recycled pure metallic gold particles from h) after calcination. Error bars represent SD. n = 3 independent experiments.

To demonstrate the practical utility of **NAS–HBA** for gold separation, we evaluated its performance under real–world conditions, beginning with catalytic wastewater sourced from a gold catalysis laboratory. The wastewater, initially containing ≈121.4 ppm gold, was treated with NaOH to adjust the pH to 1. The solution also contained substantial concentrations of competing cations, including Cu^2^+ (102 ppm) and Al^3^+ (32 ppm) (Table S3; Figure S53, Supporting Information). Exposure of 4.0 mg of **NAS–HBA** to this solution for 300 s yielded remarkable results. ICP–MS analysis revealed a dramatic reduction in gold concentration to just 831.5 ppb, corresponding to an impressive gold recovery efficiency of 99.3% (**Figure** **5**a). Crucially, no significant adsorption of competing ions was observed, demonstrating **NAS–HBA**’s exceptional selectivity and efficiency for gold recovery from such complex matrices.

The efficient recovery of gold from *aqua regia* leachates of electronic waste, such as discarded computer processing units (CPUs) and printed circuit boards (PCBs), remains a significant challenge due to their highly acidic and ion–rich nature. To address this, **NAS–HBA** was applied to leachates derived from AMD CPUs and mobile phone PCBs, processed with *aqua regia* and subsequently adjusted to pH 1 using NaOH. The initial gold concentrations were determined to be 3.6 ppm for AMD CPUs and 2.5 ppm for mobile phone PCBs (**Figure** 5b,c; Table S3, Supporting Information). Post–treatment with **NAS–HBA** reduced the residual gold content to just 35.5 ppb and 12.4 ppb, respectively, achieving over 99% gold recovery efficiency for both cases. Despite the leachates containing competing cations like Cu^2^+ at concentrations up to 2000 times higher than gold, no significant interference was observed, highlighting the remarkable selectivity of **NAS–HBA** for gold recovery (Figures S54 and S55; Table S3, Supporting Information).

To further evaluate **NAS–HBA**’s performance, we investigated its application to complex gold ore leachates. Gold ore samples, generously supplied by a metallurgical company in Jiangxi, China, were processed using *aqua regia* as a safer alternative to hazardous cyanide–based leaching methods. After removing solid residues, the resulting leachate, adjusted to pH 1 using NaOH, was found to contain only 1.6 ppm gold (**Figure** 5d). The leachate also included a broad spectrum of competing metallic ions, such as Fe^3^^+^ (14 065 ppm), Cu^2^^+^, Al^3^^+^, and Zn^2^^+^, presenting a formidable challenge for selective gold recovery (Table S3; Figure S56, Supporting Information). Remarkably, upon treatment with **NAS–HBA**, the gold concentration was reduced to just 131.1 ppb, achieving an impressive gold recovery efficiency of 91.7% (**Figure** 5d). Minimal extraction of competing ions was observed, underscoring **NAS–HBA**’s exceptional selectivity and efficiency. To the best of our knowledge, the performance of **NAS–HBA** in the most challenging scenarios of gold separation and mining surpasses that of most, if not all, known porous sorbents, including MOFs and COFs.

The extraction of gold from seawater has garnered increasing attention due to dwindling terrestrial gold reserves and the urgent need for environmentally sustainable extraction methods. However, this endeavor faces formidable challenges, as gold exists in seawater at ultra–trace levels (parts per trillion, ppt), masked by an overwhelming abundance of competing ions. The development of highly selective and efficient extraction technologies capable of processing large volumes of seawater is therefore of paramount importance.

To evaluate the potential of **NAS–HBA** for direct gold separation from natural water bodies, we conducted adsorption experiments using water samples from the Xiangjiang River and the Yellow Sea. These samples were acidified to pH 1 with HCl, and the gold concentration was adjusted to 10 ppb using NaAuBr_4_. **NAS–HBA** was then introduced to the samples, and the residual gold concentrations were monitored over time using ICP–MS. Remarkably, despite the extremely low initial gold concentrations, **NAS–HBA** achieved a reduction in gold content to 0.2 ppb for the Xiangjiang River and less than 0.1 ppb for the Yellow Sea within 30 min (**Figure** 5e,f). Notably, the removal of competing ions, including Na, Mg, Si, K, Ca, Cu, Rb, Ba, and W, was negligible, underscoring the exceptional selectivity of **NAS–HBA**. These results highlight the robust specificity and high efficiency of the superphane–based **NAS–HBA** for separating trace amounts of gold from natural water sources. This breakthrough underscores its potential as a transformative solution for gold recovery from seawater, addressing critical environmental and resource challenges.

The extraordinary capability of **NAS–HBA** to selectively and efficiently recover gold from complex real–world matrices was further validated through the extraction of high–purity metallic gold from discarded mobile phone printed circuit boards (PCBs). Secondary PCBs, sourced from an e–waste recycling facility, underwent preliminary treatment with N–bromosuccinimide (NBS) and pyridine (Py), following established protocols.^[^ ^29^ ^]^ The resulting leachate contained ≈1.8 ppm of Au^3^+ (or AuBr₄⁻) alongside significant concentrations of competing cations, including 106 ppm of Cu^2^^+^, 1.4 ppm of Ni^2^^+^, and 1.1 ppm of Pb^2^^+^ (Table S3, Supporting Information). A single one–hour treatment with **NAS–HBA** achieved near–complete gold recovery, reducing the residual gold concentration from 1.8 ppm to just 4.0 ppb (**Figure** 5g). Remarkably, this process exhibited minimal extraction of competing ions, demonstrating the exceptional selectivity of **NAS–HBA**. Building on this success, a scaled–up gold adsorption experiment was performed to recover pure gold from discarded mobile phone PCBs. **NAS–HBA** effectively extracted gold from the leachate with negligible interference from competing ions (**Figure** 5h; Table S4, Supporting Information). Following calcination, 32 mg of high–purity gold, equivalent to 23.8 Karat, was successfully recovered (**Figure** 5i). This highlights **NAS–HBA**’s potential for scalable and environmentally friendly applications in resource recycling.

## 3 Conclusion

In summary, this study reports the serendipitous discovery of a covalent organic superphane cage (**NAS–HBA**) as a nonporous amorphous sorbent, uniquely capable of capturing linear AuBr₂⁻ or AuCl₂⁻—rare and metastable intermediates derived from the more prevalent AuBr₄⁻ or AuCl₄⁻ species. **NAS–HBA** exhibits extraordinary efficiency in selectively capturing ultra–trace gold from complex aquatic environments, characterized by its exceptional gold uptake capacity (2750 mg g⁻¹), rapid adsorption kinetics (40 s), and remarkable selectivity (>99%). Extensively validated through comprehensive analytical techniques, **NAS–HBA** demonstrates unparalleled durability across a broad pH spectrum (1–11), including extreme acidic conditions (up to 6 M acids) where it achieves gold uptake capacities as high as 821 mg g⁻¹ in 36 wt.% HCl aqueous solutions. This material outperforms conventional porous sorbents in diverse applications, including the recovery of gold from e–waste, seawater, and gold ores. A highlight of this work is the successful recovery of high–purity gold (23.8 Karat) from the leachates of discarded mobile phone PCBs, showcasing the scalability and environmental sustainability of **NAS–HBA**. Beyond addressing the impending depletion of terrestrial gold resources, this breakthrough establishes a new benchmark for innovative sorbent technologies, offering a transformative and sustainable approach to gold separation. **NAS–HBA** represents a pivotal advancement with the potential to reshape methodologies in resource extraction and beyond, heralding a significant milestone in the pursuit of sustainable mining and recovery strategies.

## Conflict of Interest

Hunan University has applied one patent based on this work.

## Supporting information



Supporting Information

## Data Availability

The data that support the findings of this study are available in the supplementary material of this article.
