# Supplementary material for: Ultra–Efficient and Selective Gold Separation via Second–Sphere Coordination of Aurous Dihalide Using a Nonporous Amorphous Superadsorbent
Source: Adv Sci (Weinh). 2025 Mar 5;12(16):2501397. doi: 10.1002/advs.202501397 (PMC12021077; doi:10.1002/advs.202501397)
Supplement: Supplementary file 1 — Supporting Information [file ADVS-12-2501397-s001.pdf]

ADVANCED SCIENCE
Open Access

Supporting Information

for *Adv. Sci.*, DOI 10.1002/advs.202501397

Ultra–Efficient and Selective Gold Separation via Second–Sphere Coordination of Aurous Dihalide Using a Nonporous Amorphous Superadsorbent

*Wei Zhou, Xiao Cai, Yiyao Xu, Min Zhou, Jialian Li, Qiang Liu and Qing He**

Supporting Information

For

Ultra–Efficient and Selective Gold Separation via Second–Sphere Coordination of Aurous Dihalide Using a Nonporous Amorphous Superadsorbent

Wei Zhou,[a] Xiao Cai,[a] Yiyao Xu,[a] Min Zhou,[a] Jialian Li,[a] Qiang Liu,[a] and Qing He*,[a]

[a]State Key Laboratory of Chemo/Biosensing and Chemometrics, College of Chemistry and Chemical Engineering, Hunan University, Changsha 410082, P. R. China.

*Correspondence: heqing85@hnu.edu.cn

Contents

1. General information

2. Experimental methods

3. The crystal structure of the $Br^{-}\cdot 2H_2O$@NAS–HBA$\cdot 2H^{+}\cdot AuBr_2^{-}$ complex

4. Adsorption of gold from aqueous solutions

5. X–ray experimental details

6. References

1. General information

All solvents and chemicals were procured from Sigma–Aldrich, TCI, Energy–Chemical, or Acros and were used as received, without further purification. Thin–layer chromatography (TLC) analyses utilized Sorbent Technologies silica gel sheets (200 mesh), while flash column chromatography employed silica gel (300–400 mesh). Fourier–transform infrared (FTIR) spectra were obtained using a Shimadzu IRSpirit, with a wavenumber range of 500 cm−1 to 4000 cm^{-1}. Scanning electron microscopy (SEM) imaging of the complex was conducted on a MIRA3 LMH EDS: One Max 20. Gold adsorption analyses via inductively coupled plasma mass spectrometry (ICP–MS) were performed using an Agilent 8900, and ion chromatography data were obtained with a Thermo Fisher DIONEX ICS–600. Powder X–ray diffraction (PXRD) patterns were measured on a D8 ADVANCE X–ray diffractometer using Cu Kα radiation (λ = 1.5406 Å) across a 2–theta range of 5°–80° with a 0.2° step increment. Liquid ^{1}H NMR spectra in $CDCl_3$ and DMSO–d_6 were recorded at 400 MHz using a Bruker Avance Neo 400 spectrometer. X–ray photoelectron spectroscopy (XPS) spectra were collected on a Shimadzu AXIS SUPRA+, equipped with a monochromatic Al Kα X–ray source under ultra–high vacuum conditions. Nitrogen (N_2) sorption isotherms at 77 K were measured volumetrically using a JW–BK200C, and Brunauer–Emmett–Teller (BET) surface areas were calculated from these N2 isotherms. Raman spectra were recorded using an InVia–Reflex, NIR Raman spectrometer with a 710 nm laser. X–ray crystallographic analyses were conducted on a Bruker D8 Venture diffractometer with a μ–focused Cu Kα radiation source (λ = 1.5418 Å).

2. Experimental methods

Gold adsorption efficiency from aqueous media

The adsorbent **NAS–HBA** (4.0 mg) was immersed in an Au(III) aqueous solution, with or without the presence of competing ions such as Mg^{2+}, Al^{3+}, Cr^{3+}, Fe^{3+}, Co^{2+}, Ni^{2+}, Cu^{2+}, Zn^{2+}, Cd^{2+}, Pb^{2+}, Cl^-, Br^-, NO_4^-, SO_4^{2-} and PO_4^{3-}. The solution's composition was continuously monitored using ICP–MS and ion chromatography. The removal efficiency of gold by the adsorbent was calculated using the following equation:

Gold removal efficiency = $\frac{C_0 - C_t}{C_0} \times 100\%$

Where C_0 (ppm) and C_t (ppm) are the concentrations of aqueous Au (III) before and after adsorption, respectively.

Au (III) adsorption capability from aqueous media

The adsorbent (2.5 mg) was immersed in an aqueous Au (III) solution with varying concentrations (10–2000 ppm, 4 mL, pH=1) and stirred at room temperature for 24 hours. After filtration, the volume was adjusted to 10 mL with water. The residual concentration was then determined using ICP–MS to calculate the adsorption capacity.

Gold adsorption capability = $\frac{(C_0 - C_t * 10/4) * 4}{2.5}$

Where C_0 (ppm) and C_t (ppm) are the concentrations of aqueous Au (III) before and after adsorption, respectively.

Gold adsorption capability from different hydrogen ion concentration aqueous media

The adsorbent (2.5 mg) was immersed in a hydrogen ion concentration aqueous Au (III) solution (1600 ppm, 4 mL) and stirred at room temperature for 24 hours. After filtration, the volume was adjusted to 10 mL with water. The residual concentration of Au (III) was then determined using ICP–MS to calculate the adsorption capacity.

$$\text{Gold adsorption capability} = \frac{(1600 - C_t * 10/4) * 4}{2.5}$$

where C_t (ppm) are the concentrations of aqueous Au (III) before and after adsorption, respectively.

Material pre–treatment in the real system

Catalytic wastewater

Process: Different batches of *aqua regia* solution, with high Au (III) content from the gold catalytic laboratory, were neutralized to a pH of 1 by adding 4 M NaOH, followed by the addition of deionized water. The concentration of metal elements in these solutions was then analyzed using ICP–MS, and the samples were set aside.

Adsorption: 4 mg of **NAS–HBA** was added to a 4 mL catalytic wastewater and stirred for 5 minutes. After filtration, **NAS–HBA** was thoroughly washed with deionized water, and the volume was adjusted to 10 mL. The residual concentration was then measured using ICP–MS to calculate the adsorption capacity.

E–wastes

Process: 1) E–waste components such as CPUs or PCBs were immersed in a 10 M NaOH solution for one day to remove the epoxy coating from their surfaces. After removal, they were rinsed with tap water. The top cover of the CPU was then detached, and the exposed components were soaked in an *aqua regia* solution (concentrated HCl: concentrated HNO_3 = 3:1, v/v). The temperature of this solution was maintained at 40 ^oC for two days. Subsequently, the E–waste was removed, and the acidic solution was filtered to eliminate any undissolved residues. To adjust the pH to 1, 4M NaOH was added, followed by deionized water. The concentration of metal elements was then determined using ICP–MS and the samples were set aside. 2) The leaching solution was prepared using N–bromosuccinimide (NBS) and pyridine (Py) according to the method described by Li et al. [1]

Adsorption: In the experiment, 8 mg of **NAS–HBA** was added to 4 mL of PCB leachate solution and stirred for 5 minutes. After filtration, the **NAS–HBA** was washed extensively with deionized water and the volume was brought up to 10 mL. The residual concentration was determined using ICP–MS to calculate the adsorption amount.

Scaled–up adsorption (PCB): In the procedure, 1 g of **NAS–HBA** was added to approximately 1 L of PCB leachate solution and stirred for 5 minutes. After filtration, **NAS–HBA** was thoroughly washed with deionized water, and the volume was adjusted back to 1 L. The residual concentration was then determined using ICP–MS to calculate the adsorption amount.

Gold ores

Process: Gold ore was treated with aqua regia in a 500 mL round–bottom flask equipped with a three–way valve. Specifically, 100 g of gold ore powder was added to 80 mL of aqua regia and stirred at 600 rpm for 24 hours. After the reaction time elapsed, the leaching solution was filtered using filter paper. Then, 4 M NaOH was added to adjust the pH to 1, and deionized water was added to bring the volume to 250 mL. The solution was subsequently passed through a 0.22 μm membrane filter. The concentration of metal elements was then determined by ICP–MS and the samples were set aside.

Adsorption: In this experiment, 10 mg of **NAS–HBA** was added to a 4 mL solution of gold ores and stirred for 5 minutes. After filtration, **NAS–HBA** was thoroughly washed with deionized water, and the volume was adjusted to 10 mL. The residual concentration was subsequently determined using ICP–MS to assess the adsorption capacity.

Sea water and river water

Process: The pH of both sea water and river water was adjusted to 1 using HCl. Subsequently, 10 ppb of $NaAuBr_4$ was added to serve as the stock solutions.

Adsorption: 8 mg of **NAS–HBA** was added to 4 mL stock solutions of both sea water and river water, and the mixture was stirred for 30 minutes. After filtration, the **NAS–HBA** was extensively washed with deionized water, and the volume adjusted to 10 mL. The residual concentration was subsequently determined using ICP–MS to calculate the adsorption capacity.

The gold recovery of Au@NAS–HBA

After adsorbing catalyzed wastewater and PCBs, the complex (Au@**NAS–HBA**) was placed in a muffle furnace and heated at 1000 ^{o}C for 5 hours to achieve high purity gold. Subsequently, a specified amount of gold was weighed, dissolved in *aqua regia*, and calibrated. The purity of the gold was then determined using ICP–MS.

Regeneration and recycling experiment

The complex Au@**NAS–HBA** was immersed in a 25 mL solution containing 5 wt% thiourea and 0.25 M K_2CO_3, and stirred at 40 ^{o}C for 4 hours. Subsequently, the mixture was filtered, and the **NAS–HBA** was extensively washed with deionized water. Chloroform was employed to dissolve the filtrate, which was then filtered; the solvent was evaporated to recover **NAS–HBA**. The stability of **NAS–HBA** was confirmed over 30 cycles. The adsorption–desorption process was repeatedly monitored using ICP–MS for 30 cycles. The purity of the recycled **NAS–HBA** was further verified by ^{1}H NMR spectroscopy in $CDCl_3$.

3. The crystal structure of the $Br^{-}\cdot 2H_2O$@NAS–HBA$\cdot 2H^{+}\cdot AuBr_2^{-}$ complex

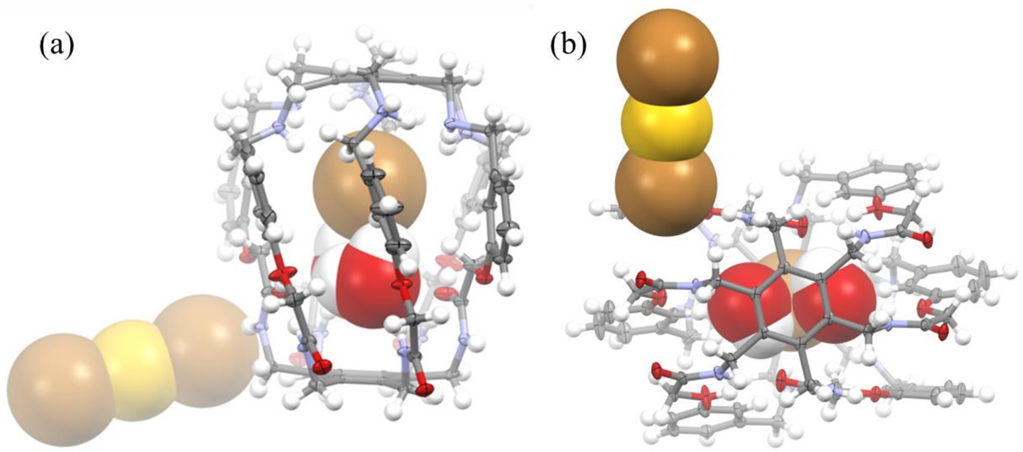

Figure S1. Single–crystal structure of $Br^{-}\cdot 2H_2O$@**NAS–HBA**$\cdot 2H^{+}\cdot AuBr_2^{-}$ complex. (a) Front view and (b) Top view.

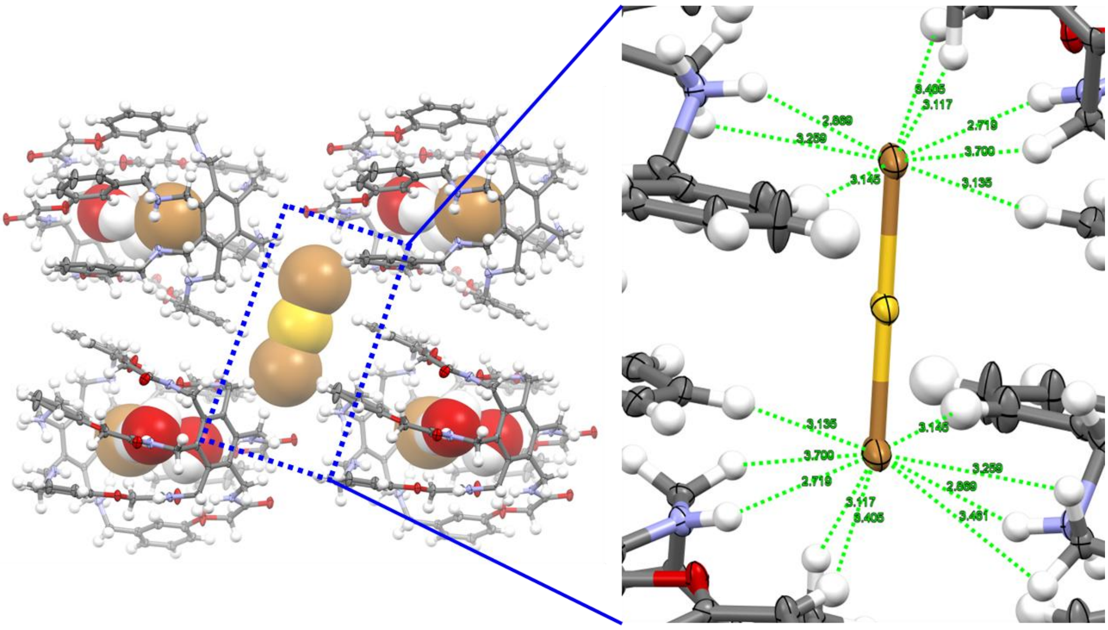

Figure S2. One selected $AuBr_2^{-}$ ions surrounded by four $Br^{-}\cdot 2H_2O$@**NAS–HBA**$\cdot 2H^{+}$ complex.The selected $AuBr_2^{-}$ interacting with four $Br^{-}\cdot 2H_2O$@**NAS–HBA**$\cdot 2H^{+}$ complexes through multiple hydrogen bonds as indicated by green dashed lines (short C/N–H···Br contacts of 2.72 – 3.46 Å).

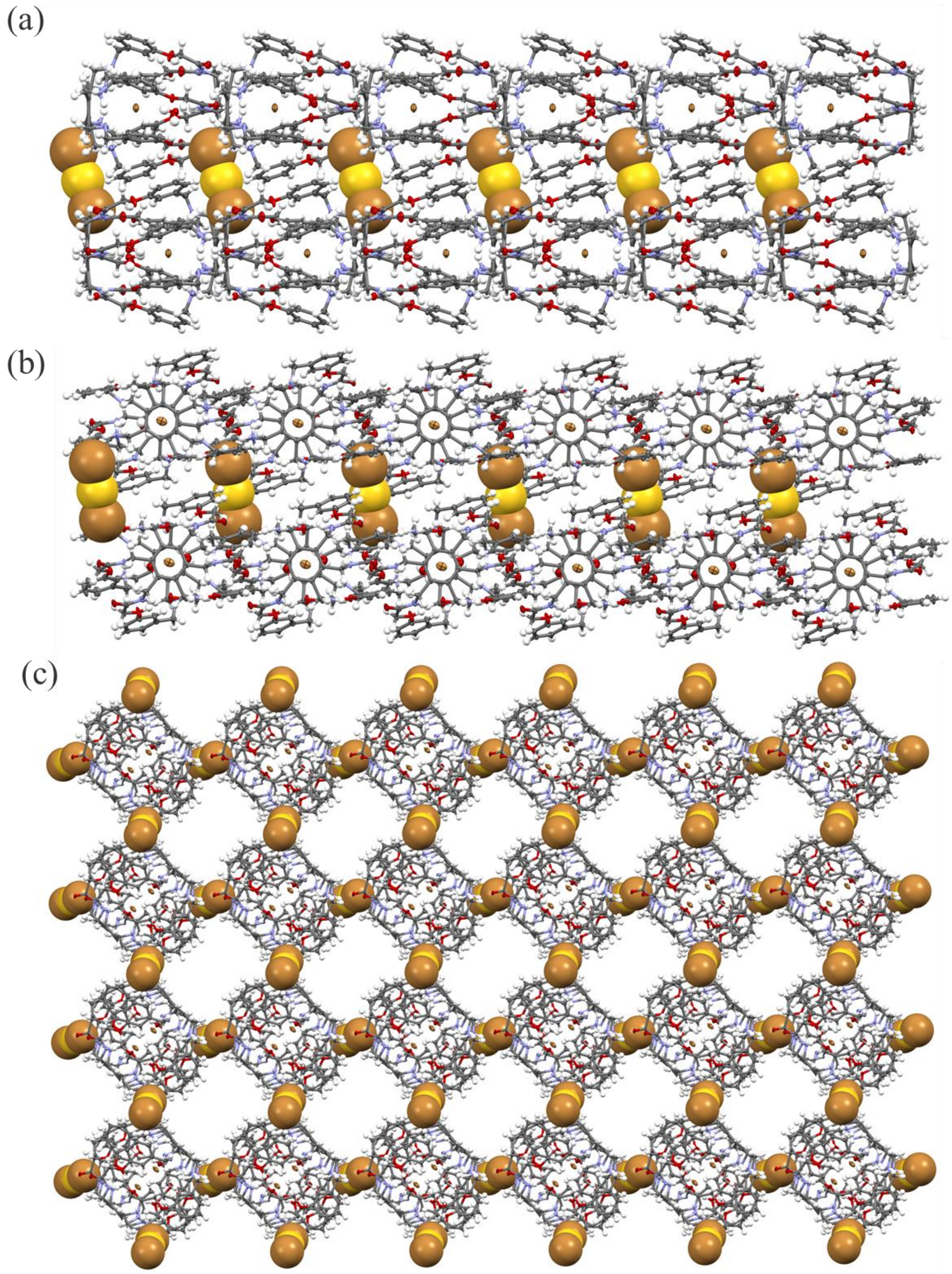

Figure S3. Different perspectives (a, b, c) on the repeating units of the two–dimensional supramolecular organic frameworks mediated by $AuBr_2^-$.

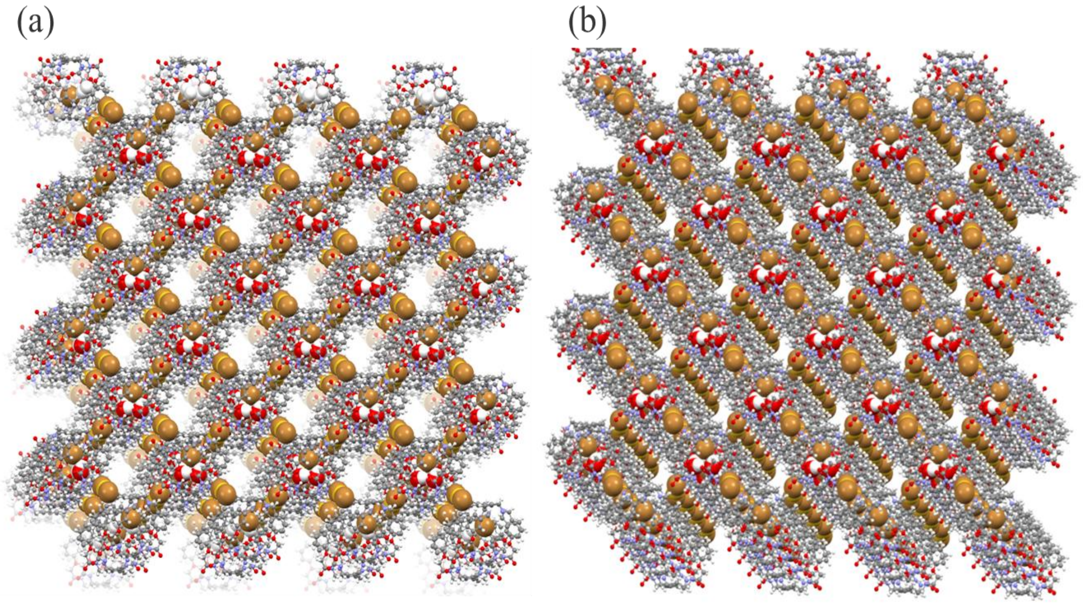

Figure S4. (a) and (b) various perspectives of the three–dimensional supramolecular organic frameworks mediated by $AuBr_2^-$.

4. Adsorption of gold from aqueous solutions

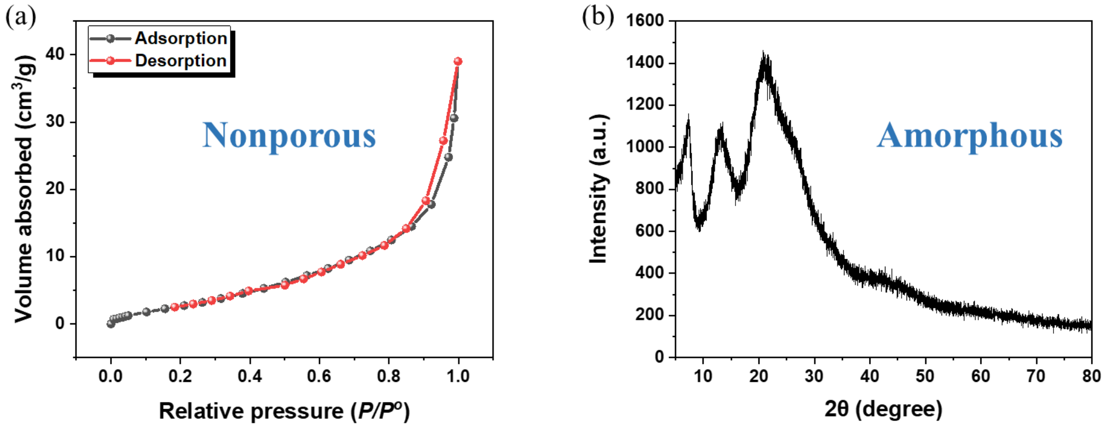

Figure S5. The characterization of **NAS–HBA**. (a) The N_2 adsorption (solid symbols)/desorption (open symbols) isotherms at 77 K and (b) Powder X–ray diffraction patterns.

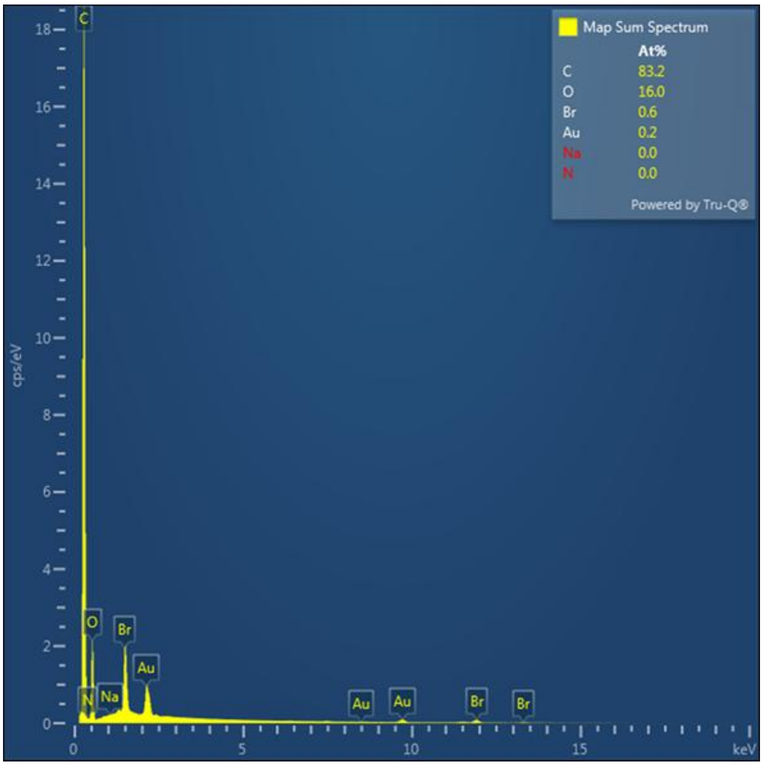

Figure S6. SEM–EDS results for **NAS–HBA** material after adsorption of $NaAuBr_4$.

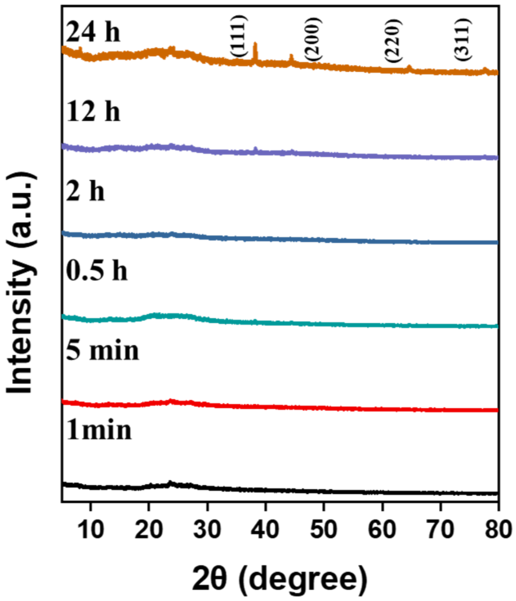

Figure S7. Evolution of the PXRD spectrum of **NAS–HBA** during the adsorption of $NaAuBr_4$.

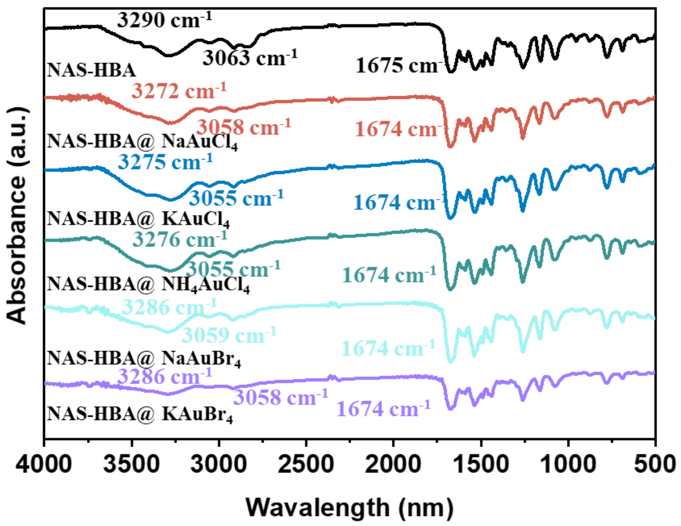

Figure S8. FTIR spectra of **NAS–HBA** only, $NaAuCl_4$@**NAS–HBA**, $KAuCl_4$@**NAS–HBA**, NH_4AuCl_4@**NAS–HBA**, $NaAuBr_4$@**NAS–HBA** and $KAuBr_4$@**NAS–HBA**.

Table S1. The pH values before and after the adsorption of $NaAuCl_4$ or $NaAuBr_4$ by the **NAS–HBA**.

Types of gold salt	Initial pH	pH after adsorption	Blank
$NaAuCl_4$	4.84	2.31	4.63
$NaAuBr_4$	5.73	4.11	5.64

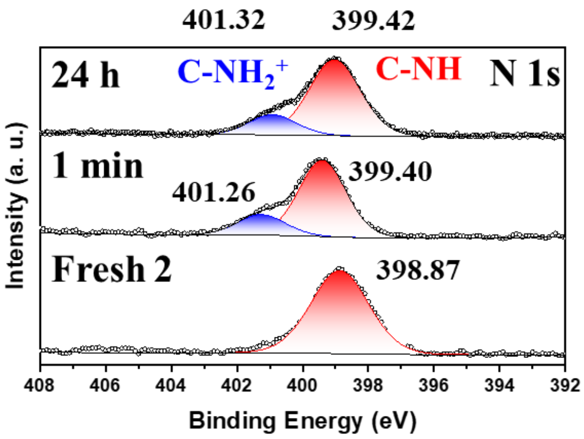

Figure S9. Evolution of the N *1s* XPS spectrum of **NAS–HBA** during the adsorption of $NaAuBr_4$.

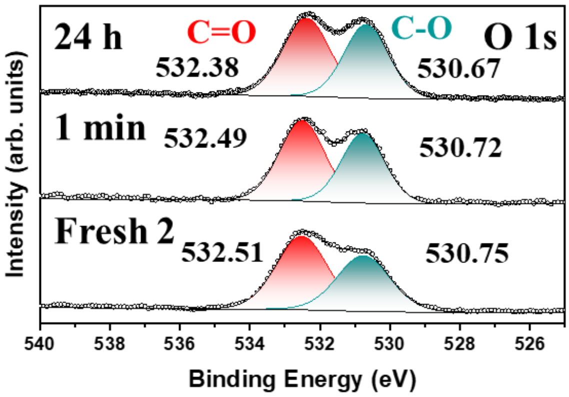

Figure S10. Evolution of the O *1s* XPS spectrum of **NAS–HBA** during the adsorption of $NaAuBr_4$.

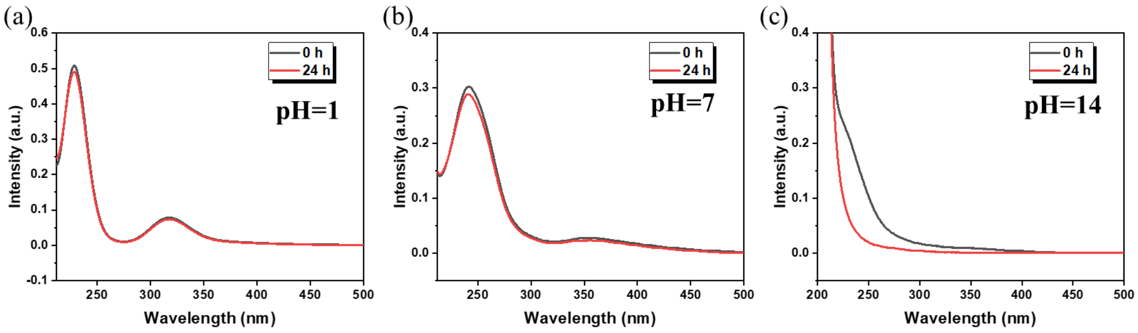

Figure S11. Time–resolved UV–Vis spectra of $NaAuBr_4$ aqueous solution at different pH levels: (a) pH = 1, (b) pH = 7, and (c) pH = 14.

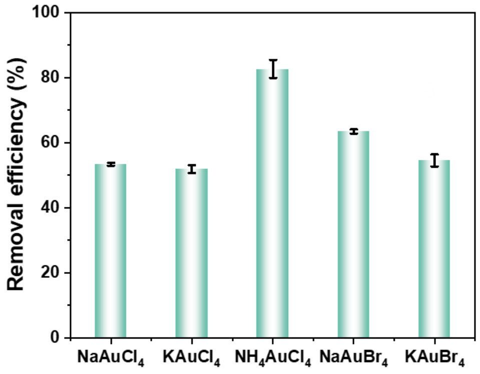

Figure S12. Adsorption efficiency of **NAS–HBA** on different gold salts at pH= 14.

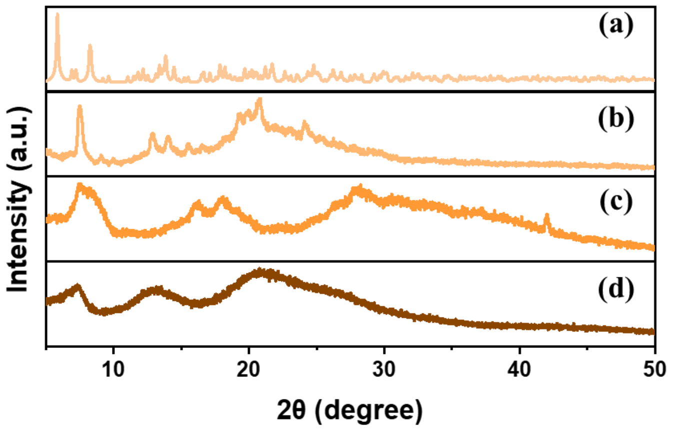

Figure S13. The PXRD spectra of simulated Br^{-}–$2H_2O$@**NAS–HBA**–$2H^{+}$ complex single crystals (a), **NAS–HBA** after adsorption of $NaAuBr_4$, blank experiment (without $NaAuBr_4$) and fresh **NAS–HBA**. The concentrations of $NaAuBr_4$ were all 1000 ppm, **NAS–HBA** was 20 mg, and the adsorption time was 30 minutes.

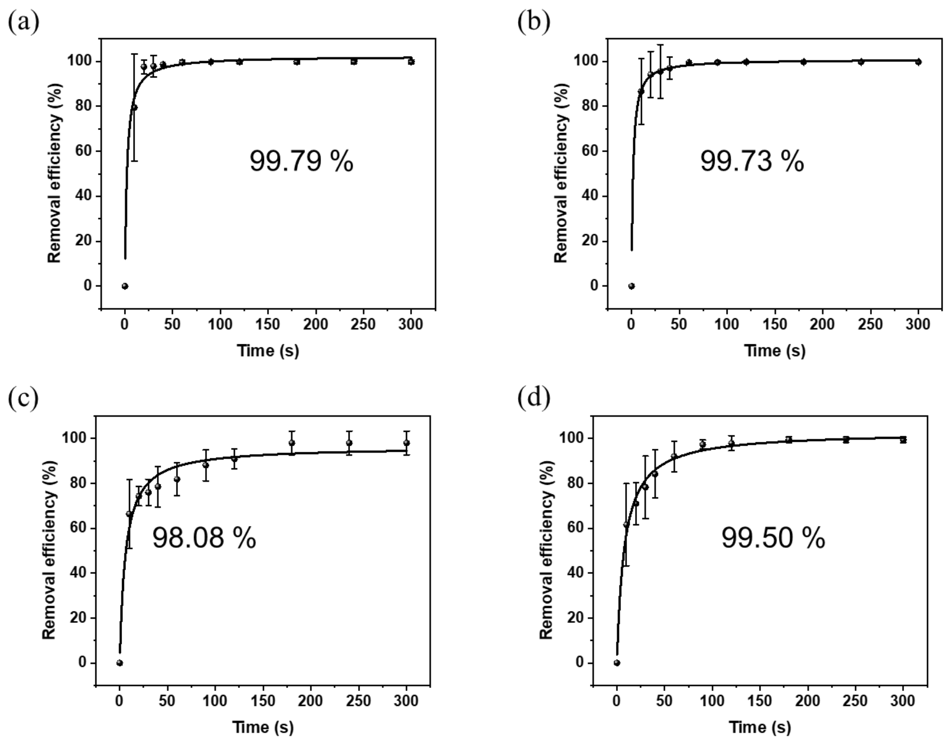

Figure S14. Time–resolved gold recovery efficacy of **NAS–HBA** from aqueous solutions of (a) $NaAuCl_4$, (b) $KAuCl_4$, (c) NH_4AuCl_4 and (d) $KAuBr_4$.

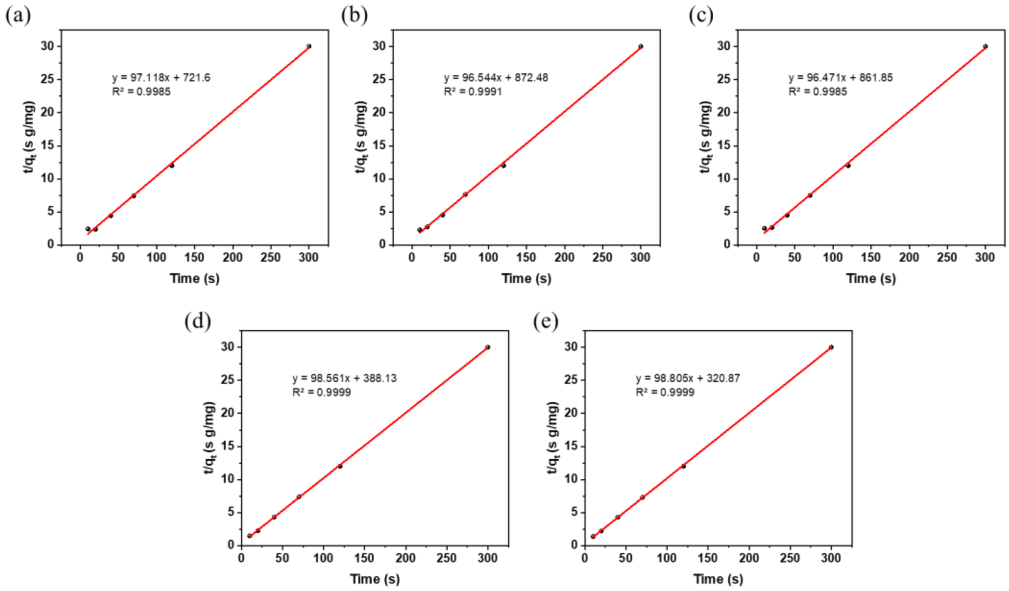

Figure S15. Pseudo–second order kinetic model of Au (III) adsorption from aqueous solutions with **NAS–HBA** at pH=1, (a) $NaAuCl_4$, (b) $KAuCl_4$, (c) NH_4AuCl_4, (d) $NaAuBr_4$ and (e) $KAuBr_4$. These data were derived from figure S14.

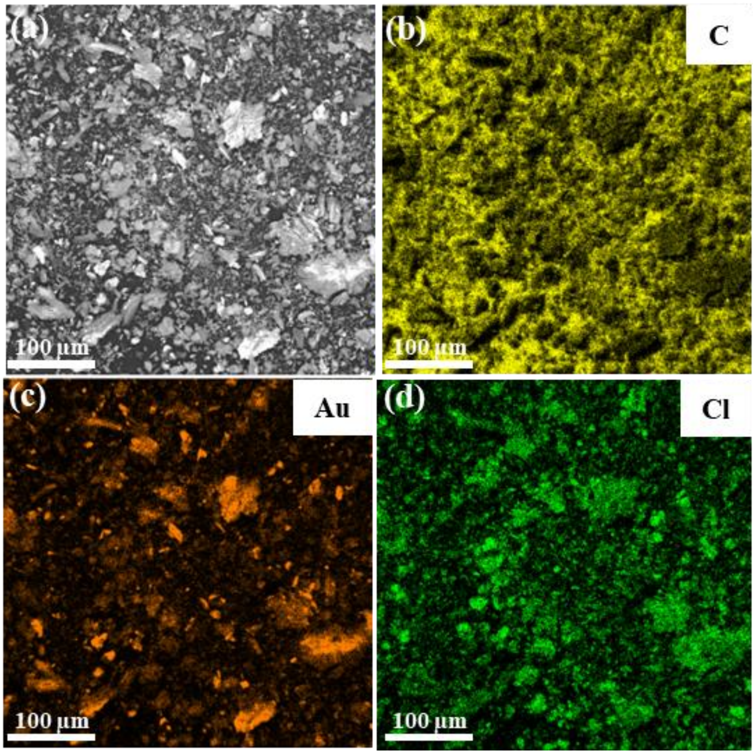

Figure S16. (a) SEM image of **NAS–HBA** after adsorption of $NaAuCl_4$ and the corresponding SEM/EDS mappings for (b) C, (c) Au and (d) Cl.

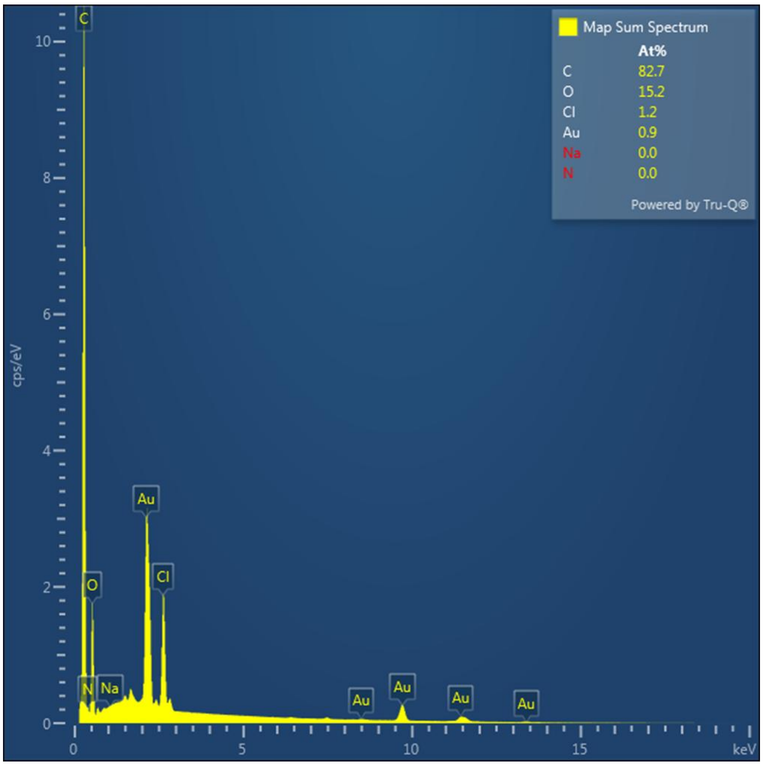

Figure S17. SEM–EDS results for **NAS–HBA** after adsorption of $NaAuCl_4$.

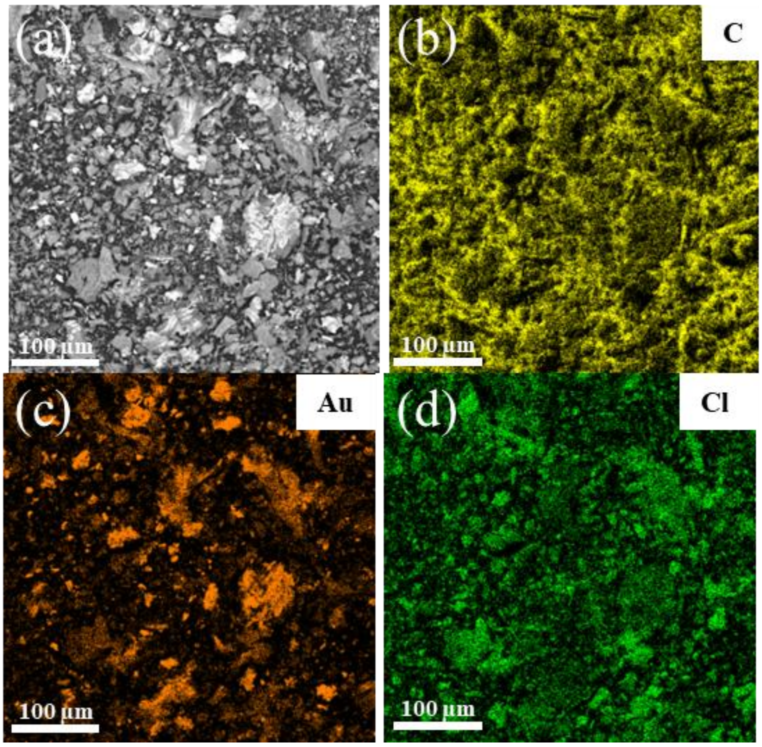

Figure S18. (a) SEM image of **NAS–HBA** after adsorption of $KAuCl_4$ and the corresponding SEM/EDS mappings for (b) C, (c) Au and (d) Cl.

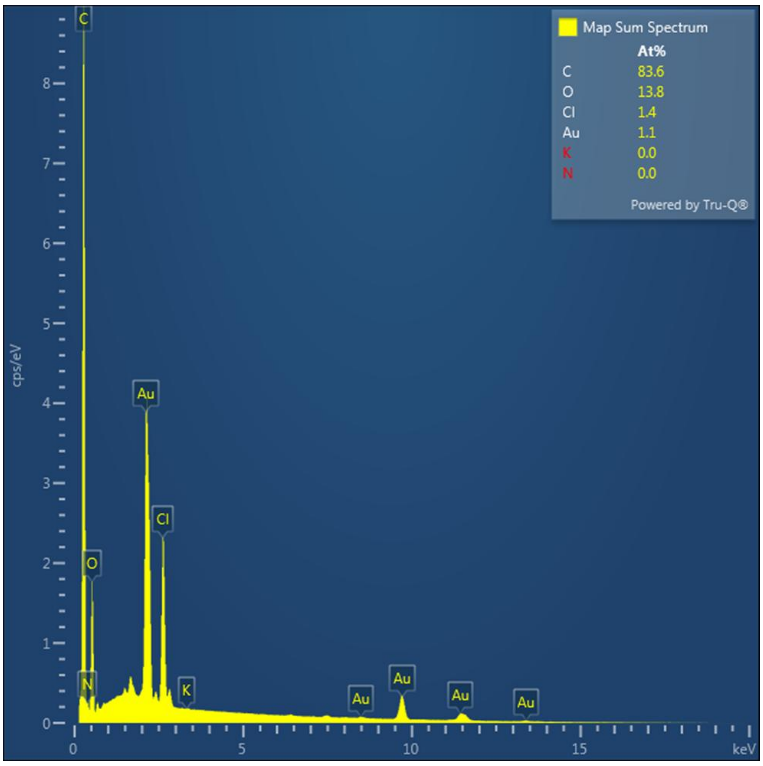

Figure S19. SEM–EDS results for **NAS–HBA** after adsorption of $KAuCl_4$.

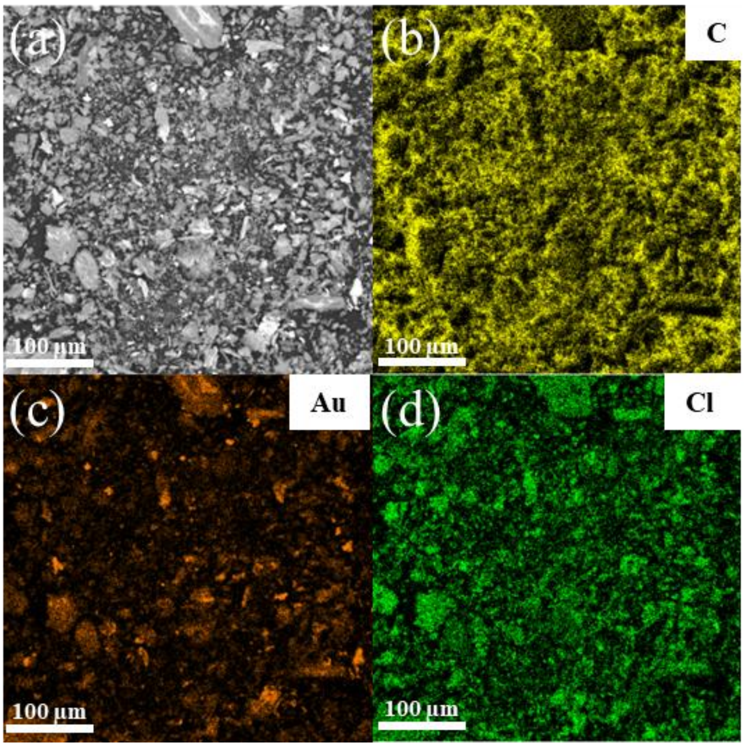

Figure S20. (a) SEM image of **NAS–HBA** after adsorption of NH_4AuCl_4 and the corresponding SEM/EDS mappings for (b) C, (c) Au and (d) Cl.

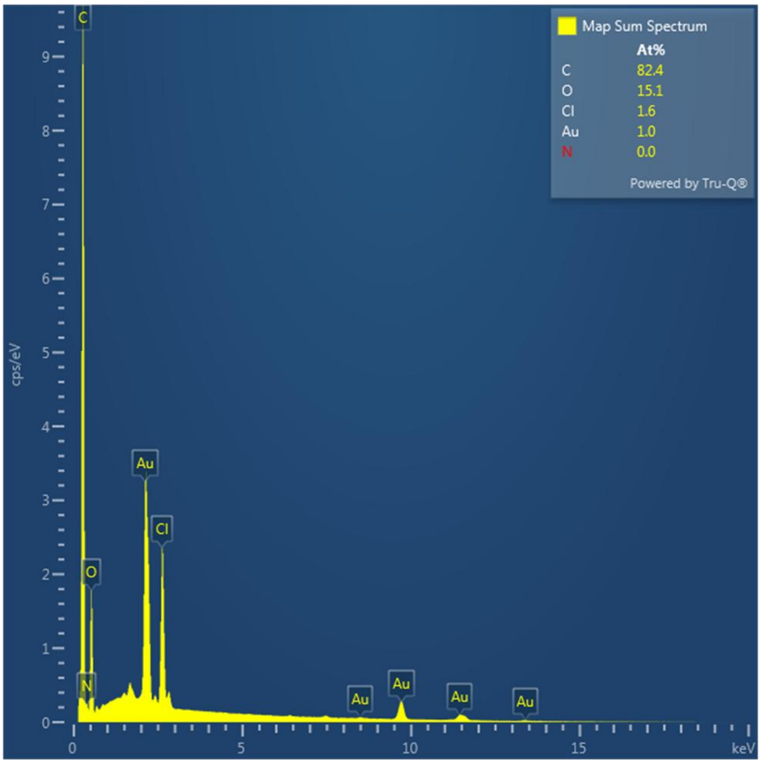

Figure S21. SEM–EDS results for **NAS–HBA** after adsorption of NH_4AuCl_4.

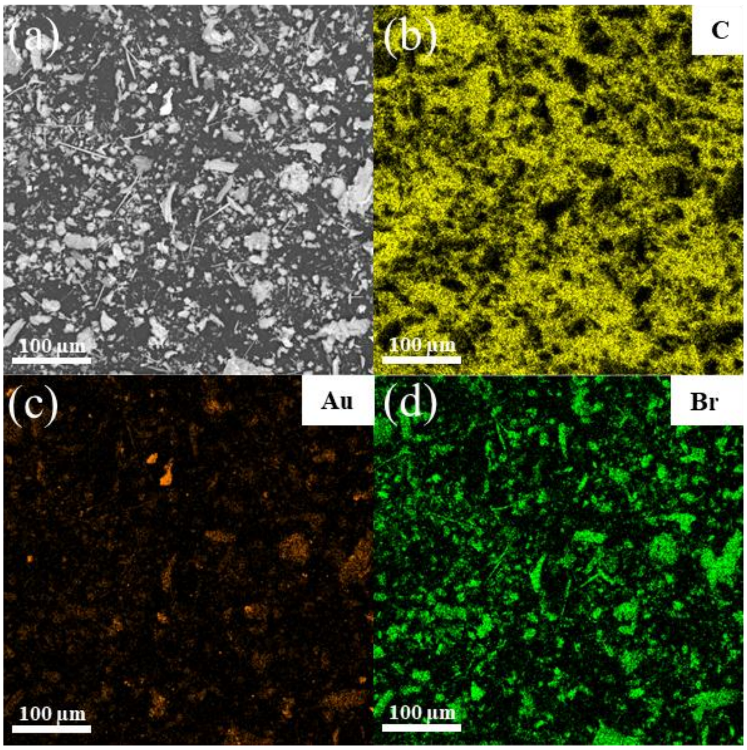

Figure S22. (a) SEM image of **NAS–HBA** after adsorption of $KAuBr_4$ and the corresponding SEM/EDS mappings for (b) C, (c) Au and (d) Cl.

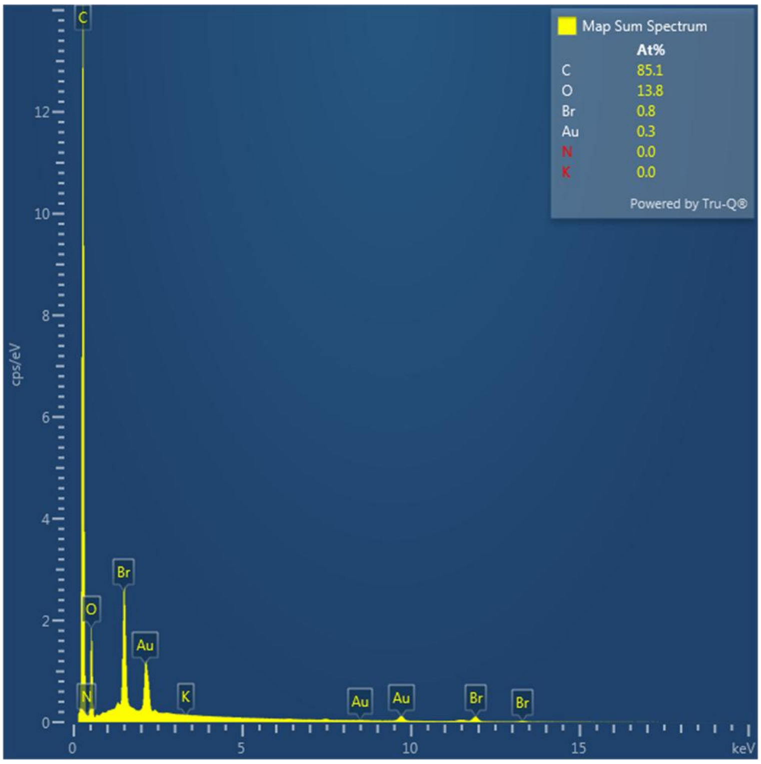

Figure S23. SEM–EDS results for **NAS–HBA** after adsorption of $KAuBr_4$.

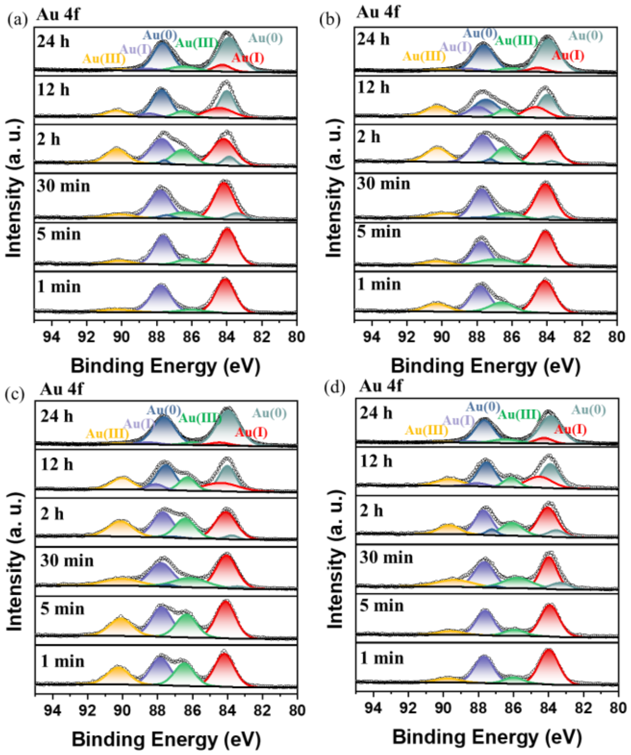

Figure S24. Evolution of the XPS spectrum of **NAS–HBA** during the adsorption of different Au(III) species at 0 min, 5 min, 10 min, 20 min and 30 min: (a) $NaAuCl_4$, (b) $KAuCl_4$, (c) NH_4AuCl_4 and (d) $KAuBr_4$.

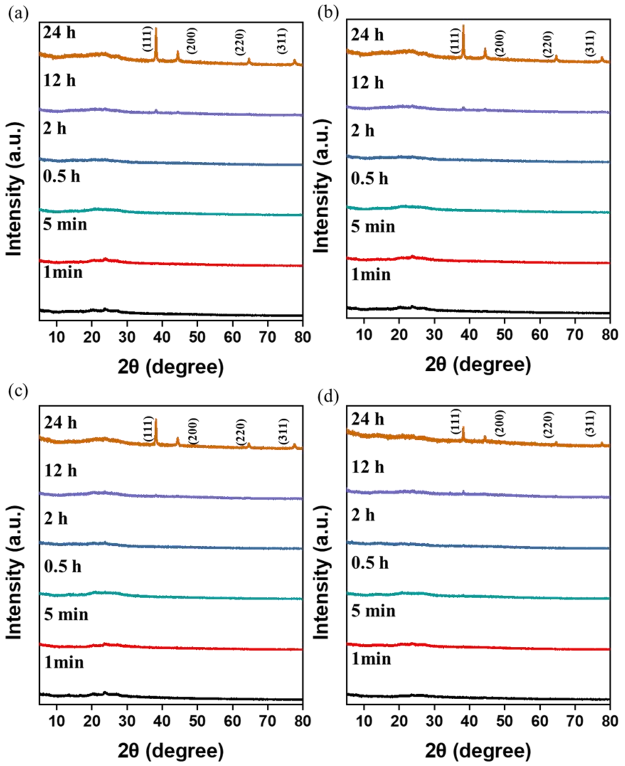

Figure S25. Evolution of the PXRD spectrum of **NAS–HBA** during the adsorption of different Au(III) species at 0 min, 5 min, 10 min, 20 min and 30 min: (a) $NaAuCl_4$, (b) $KAuCl_4$, (c) NH_4AuCl_4 and (d) $KAuBr_4$.

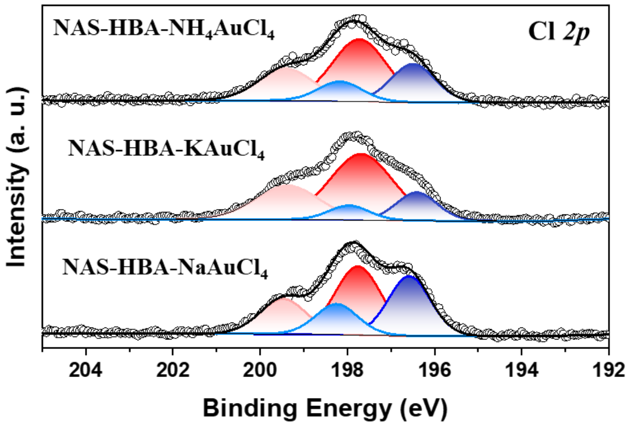

Figure S26. Cl *2p* XPS characterization of **NAS–HBA** after adsorption of $NaAuCl_4$, $KAuCl_4$ and NH_4AuCl_4.

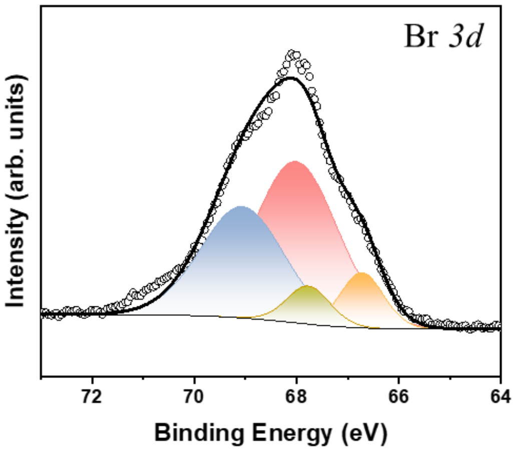

Figure S27. Br *3d* XPS characterization of **NAS–HBA** after adsorption of $KAuBr_4$.

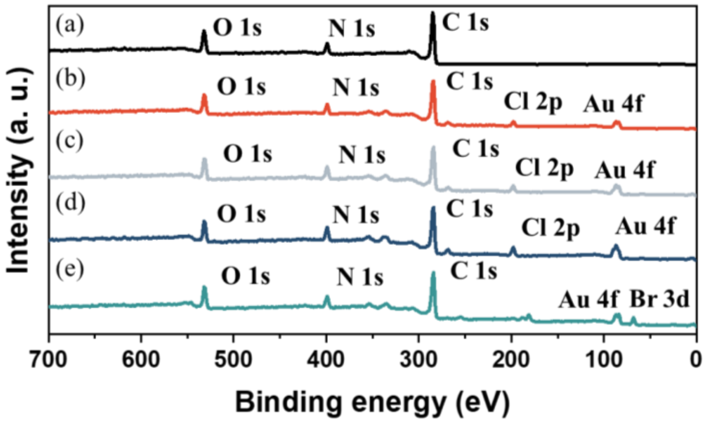

Figure S28. Full XPS spectrum of **NAS–HBA**: (a) before adsorption, and after adsorption of (b) $NaAuCl_4$, (c) $KAuCl_4$, (d) NH_4AuCl_4 and (e) $KAuBr_4$.

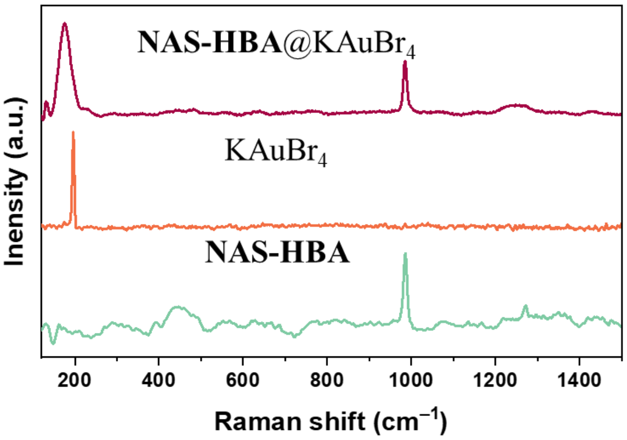

Figure S29. Full Raman spectra of **NAS–HBA** alone, $KAuBr_4$ alone, and **NAS–HBA** after adsorption of $KAuBr_4$.

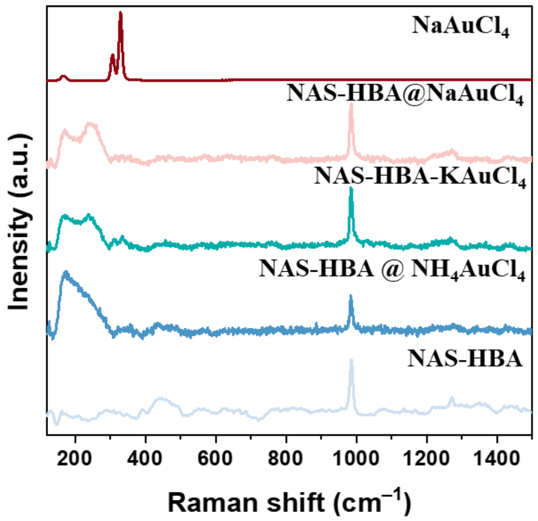

Figure S30. Full Raman spectra of **NAS–HBA** alone, $NaAuCl_4$ alone, and **NAS–HBA** after adsorption of $NaAuCl_4$, $KAuCl_4$ and NH_4AuCl_4.

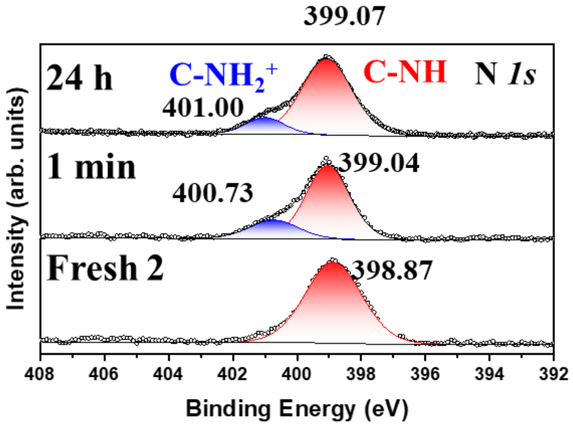

Figure S31. Evolution of the N *1s* XPS spectrum of **NAS–HBA** during the adsorption of $NaAuCl_4$ at 0 min, 1 min and 24 h.

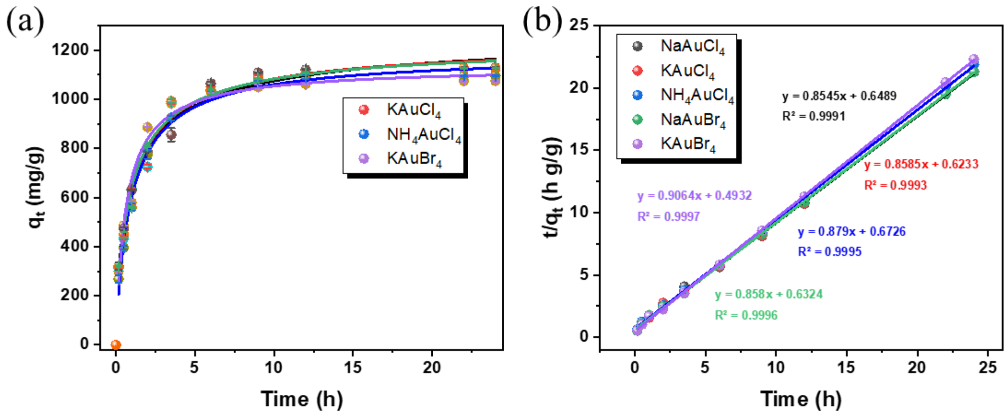

Figure S32. (a) Adsorption kinetics and (b) linear fitting using the Langmuir quasi–second–order kinetic equation for the adsorption of $NaAuCl_4$ (in black), $KAuCl_4$ (in red), NH_4AuCl_4 (in blue), $NaAuBr_4$ (in green) and $KAuBr_4$ (in purple) solutions with a concentration of 600 ppm (**NAS–HBA**, 2.5 mg; initial gold concentration: 600 mg/L; volume: 8 mL; initial pH: 1).

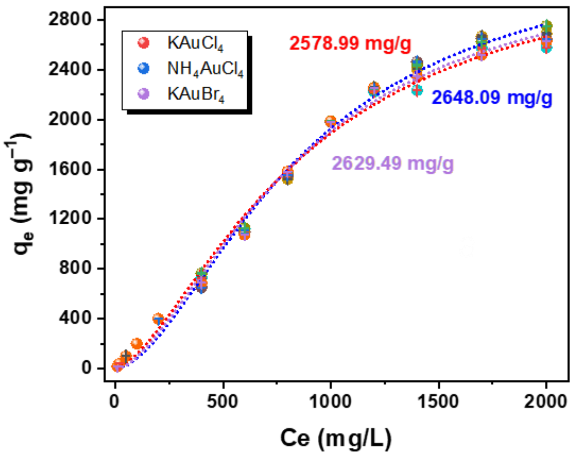

Figure S33. Maximum gold adsorption capacity as a function of adsorbate concentration, Ce is the concentration of Au(III) in aqueous solution before adsorption.

Table S2. Comparison of gold maximum adsorption capacity from aqueous phases.

Materials	Category	Mechanism	Hours (h)	Regeneration (cycles)	Selectivity	Maximum uptake Capacity (mg g^{-1})	pH	Ref.
NAS–HBA	NAS	Adsorption/ Reduction	24	√ (31)	√	2750	1	This work
NAS–HBA	NAS	Adsorption/ Reduction	24	√ (31)	√	2707	0.3 M	This work
NAS–HBA	NAS	Adsorption/ Reduction	24	√ (31)	√	2711	0.6 M	This work
NAS–HBA	NAS	Adsorption/ Reduction	24	√ (31)	√	2611	1 M	This work
NAS–HBA	NAS	Adsorption/ Reduction	24	√ (31)	√	2691	2 M	This work
NAS–HBA	NAS	Adsorption/ Reduction	24	√ (31)	√	2568	6 M	This work
NAS–HBA	NAS	Adsorption/ Reduction	24	√ (31)	√	821	12 M	This work
TpTsc–COF^g	COF	Adsorption/ Reduction	48	√ (6)	×	4400	5	[2]
S,N–rich MOF	MOF	Adsorption/ Reduction	48	√ (7)	√	3680 (55 °C)	2.57	[3]

PDA–TFN–A	POP	Adsorption/ Reduction	–	√ (5)	√	2771	2.0	[4]
PAF–1–thiourea	COF	Adsorption/ Reduction	48	√ (10)	√	2629	5	[5]
NH 2 –UiO–66	MOF	Adsorption/ Reduction	0.4	√ (10)	√	2040	2.5	[6]
cotton gel	Gel	Adsorption/ Reduction	30	×	√	1970 (55 ºC)	1	[7]
COP	POP	Adsorption/ Reduction	24	√ (5)	√	1945	2.5	[8]
PYTA–TDTA–COF	COF	Adsorption/ Reduction	12	√(10)	√	1888	2	[9]
TzDa–COF	COF	Adsorption/ Reduction	48	×	√	1866	3M	[10]
MXGA	Gel	Adsorption/ Reduction	1	×	×	1851	0.5	[11]
3D MOF@CNT	MOF	Adsorption/ Reduction	8	√ (6)	×	1832 (45 ºC)	1	[12]
MTpPa–1	COF	Adsorption/ Reduction	16	√ (15)	√	1737	2	[13]
COF–HNU25	COF	Adsorption	4	√ (20)	√	1725	6	[14]
COP–180	POP	Adsorption/ Reduction	25	√ (3)	√	1620	2	[15]
BUT–33–PpPD	MOF	Adsorption/ Reduction	0.012 5	√ (16)	√	1600	3	[16]
MOF–808@ZIF–90–XE	MOF	Adsorption/ Reduction	6	√ (5)	√	1575	5	[17]
i–POPs	POP	Adsorption/ Reduction	48	√ (8)	√	1543	6	[18]
TSC–CCB	Polymer	Adsorption/ Reduction	24	√ (3)	√	1470	6	[19]
N3–COP	POP	Adsorption/ Reduction	24	√ (5)	√	1220	4	[20]
COFJNU–1	COF	Adsorption/ Reduction	–	√ (4)	√	1124	2M	[21]

PEHA	POP	Adsorption/ Reduction	24	√ (5)	√	1086	2	[22]
TY–Hz COF	COF	Adsorption/ Reduction	0.033	√ (5)	√	1008	3	[23]
Fe–BTC/PmAP	MOF	Adsorption/ Reduction	24	√ (31)	√	934	3	[24]
NH2–CTFs	COF	Adsorption/ Reduction	12	√ (6)	√	909	3	[25]
IECS–GLA	polymer	Adsorption/ Reduction	1.5	√ (5)	√	810	3	[26]
Se–polyurethanes	polymer	Adsorption/ Reduction	24	√ (7)	√	802	–	[27]
UiO–66–MSA	MOF	Adsorption/ Reduction	24	√ (5)	√	741	5	[28]
MCPs	polymer	Adsorption/ Reduction	0.5	×	√	665	0.93	[29]
DONA–MOF	MOF	Adsorption/ Reduction	2	√ (5)	√	637	3	[30]
1	MOF	Adsorption/ Reduction	1	–	√	598	–	[31]
TTB–COF	COF	Adsorption/ Reduction	12	√(4)	√	560	1M	[32]
UiO–66–MTD	MOF	Adsorption/ Reduction	3	√ (4)	√	301	6	[33]
Magnetic nanoparticles	NM	Adsorption/ Reduction	0.5	√ (6)	√	224	3	[34]
RS–SR–NH–SiO_2–Fe_3O_4	NM	Adsorption/ Reduction	–	√ (5)	√	222	5	[35]

– : not mention.

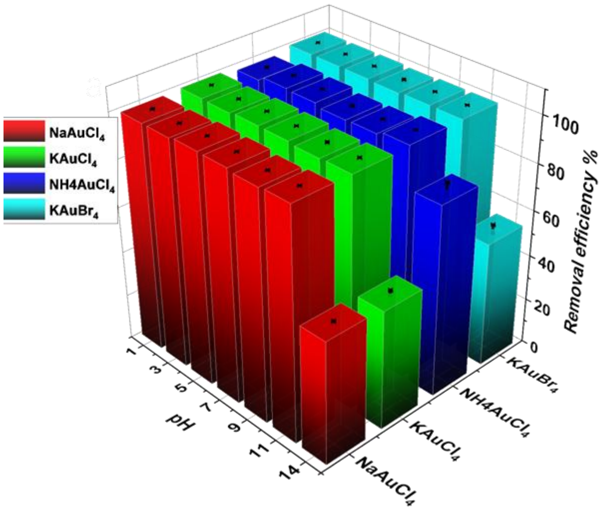

Figure S34. Effect of pH on the adsorption of $NaAuCl_4$, $KAuCl_4$, NH_4AuCl_4 and $KAuBr_4$ by **NAS–HBA**.

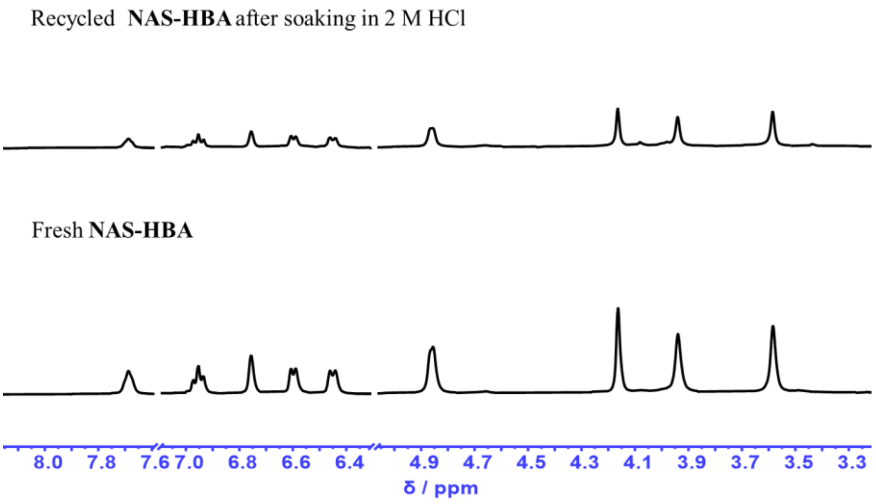

Figure S35. Partial ^1H NMR spectra (in $CDCl_3$) of fresh (bottom) and recycled (top) **NAS–HBA** after soaking for 24 h in 2 M HCl and rinsing with 0.5 M $NaHCO_3$.

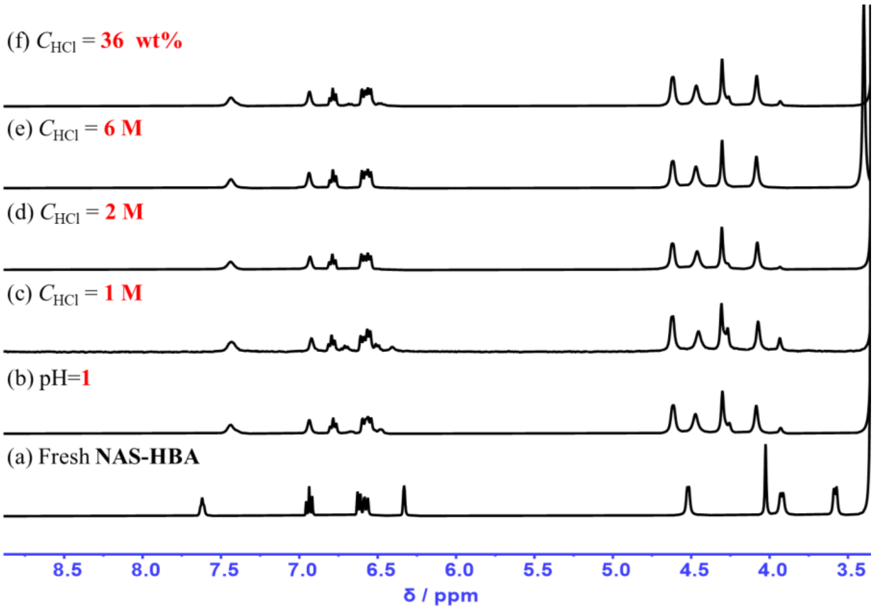

Figure S36. The partial ^{1}H NMR spectra of **NAS–HBA** stirred for 24 h in aqueous hydrochloric acid solutions at different concentrations, (a) fresh **NAS–HBA**, (b) pH=1, (c)C_{HCl}=1 M, (d) C_{HCl}=2 M, (e) C_{HCl}=6 M, (e)C_{HCl}=36 wt%.

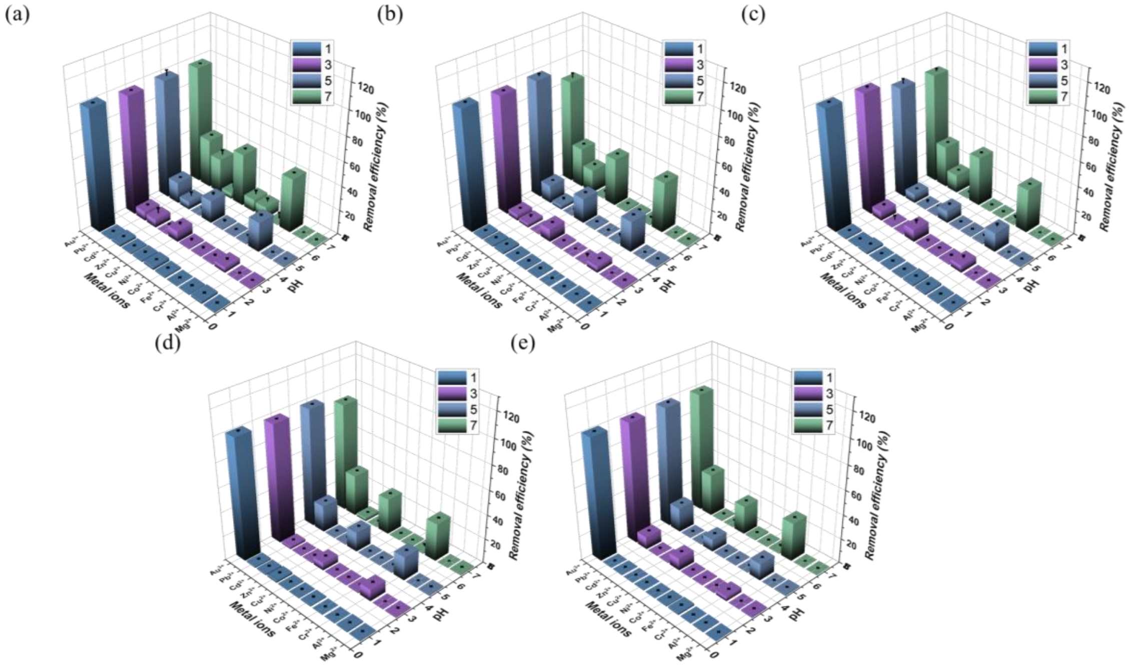

Figure S37. Adsorption of different types of Au(III) aqueous solutions containing 1 equivalent of equal–concentration competing cations (Mg^{2+}, Al^{3+}, Cr^{3+}, Fe^{3+}, Co^{2+}, Ni^{2+}, Cu^{2+}, Zn^{2+}, Cd^{2+} and Pb^{2+}) with **NAS–HBA** at pH levels of 1, 3, 5 and 7: (a) $NaAuCl_4$, (b) $KAuCl_4$, (c) NH_4AuCl_4, (d) $NaAuBr_4$ and (e) $KAuBr_4$.

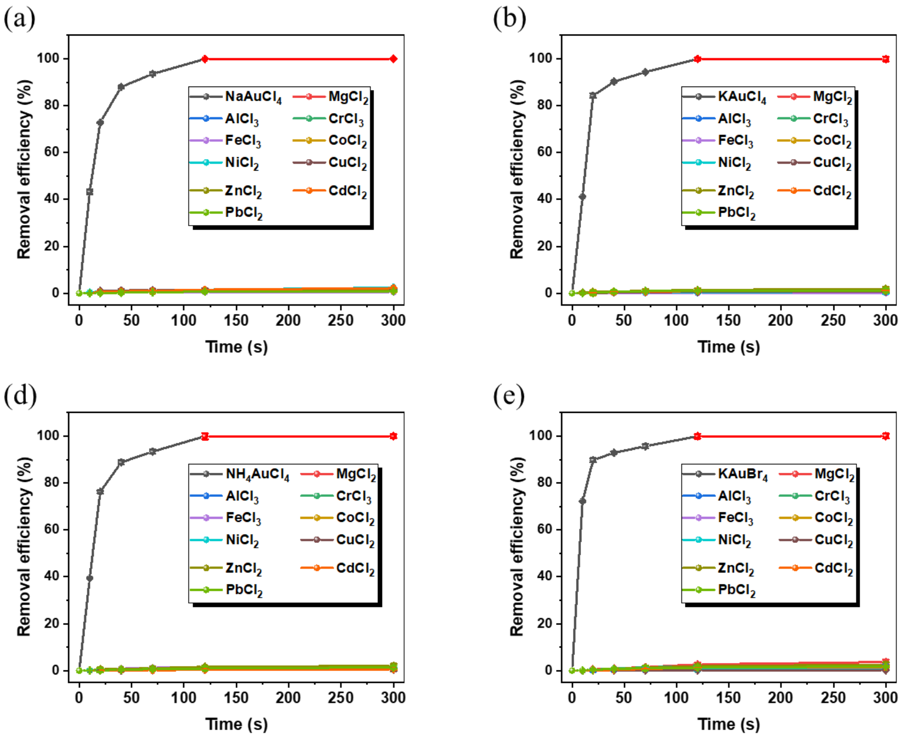

Figure S38. Adsorption of different types Au (III) aqueous solutions containing 1 equivalent of equal–molar concentration competing cations, *viz.* Mg^{2+}, Al^{3+}, Cr^{3+}, Fe^{3+}, Co^{2+}, Ni^{2+}, Cu^{2+}, Zn^{2+}, Cd^{2+} and Pb^{2+}, with **NAS–HBA** at pH=1, (a) $NaAuCl_4$, (b) $KAuCl_4$, (c) NH_4AuCl_4 and (d) $KAuBr_4$. The concentration of **NAS–HBA** was 1 mg / mL. Error bars represent SD. n=3 independent experiments.

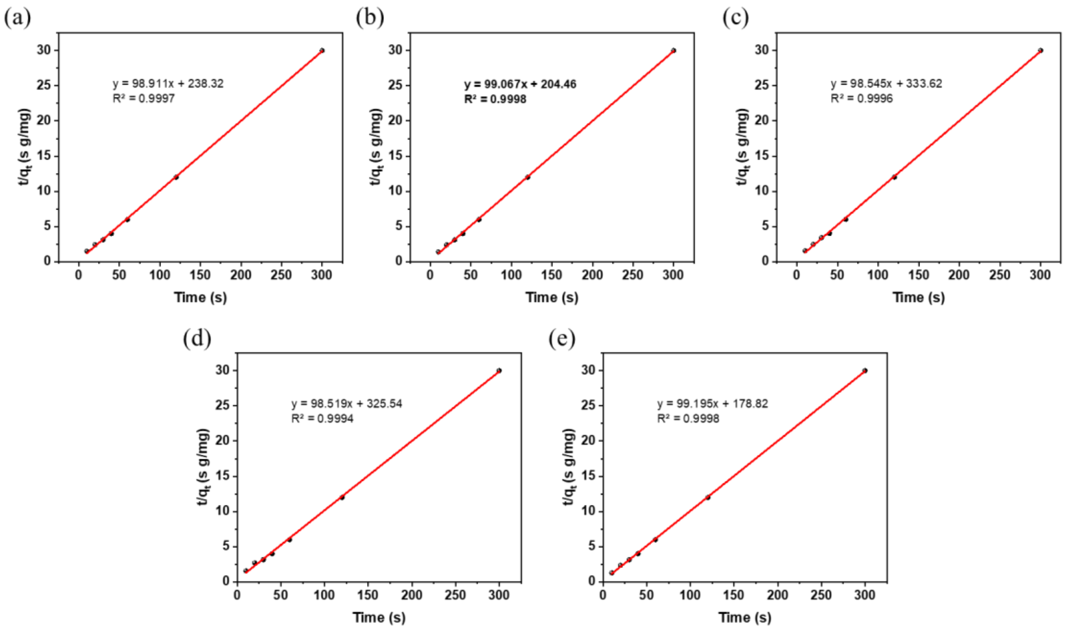

Figure S39. Pseudo–second order kinetic model of Au (III) adsorption from aqueous solutions with **NAS–HBA** at pH=1, (a) $NaAuCl_4$, (b) $KAuCl_4$, (c) NH_4AuCl_4, (d) $NaAuBr_4$ and (e) $KAuBr_4$. These data were derived from figure S38. and figure 4c.

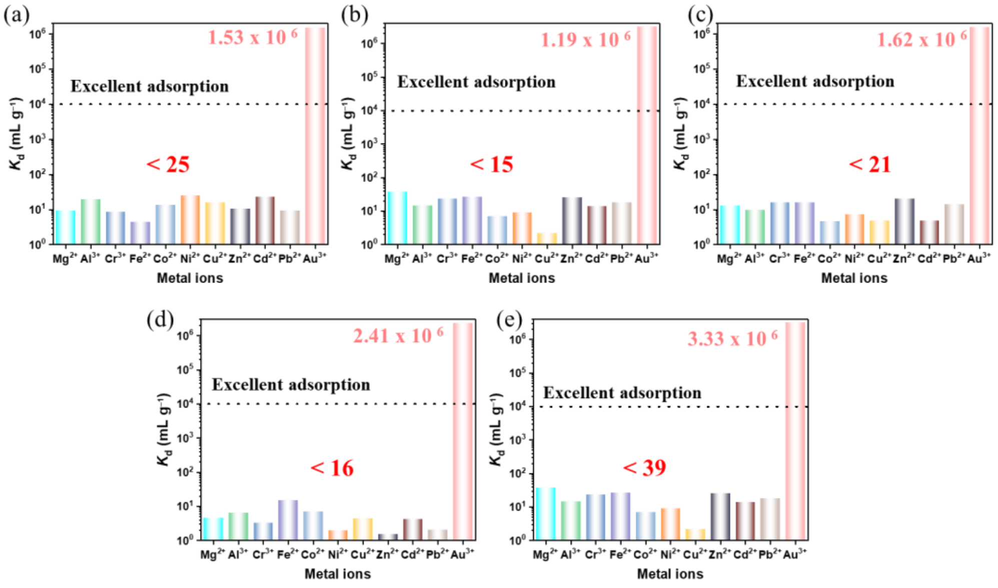

Figure S40. The distribution coefficients (K_d) of **NAS－HBA** for different metals adsorption efficiency: (a) $NaAuCl_4$, (b) $KAuCl_4$, (c) NH_4AuCl_4, (d) $NaAuBr_4$ and (e) $KAuBr_4$.

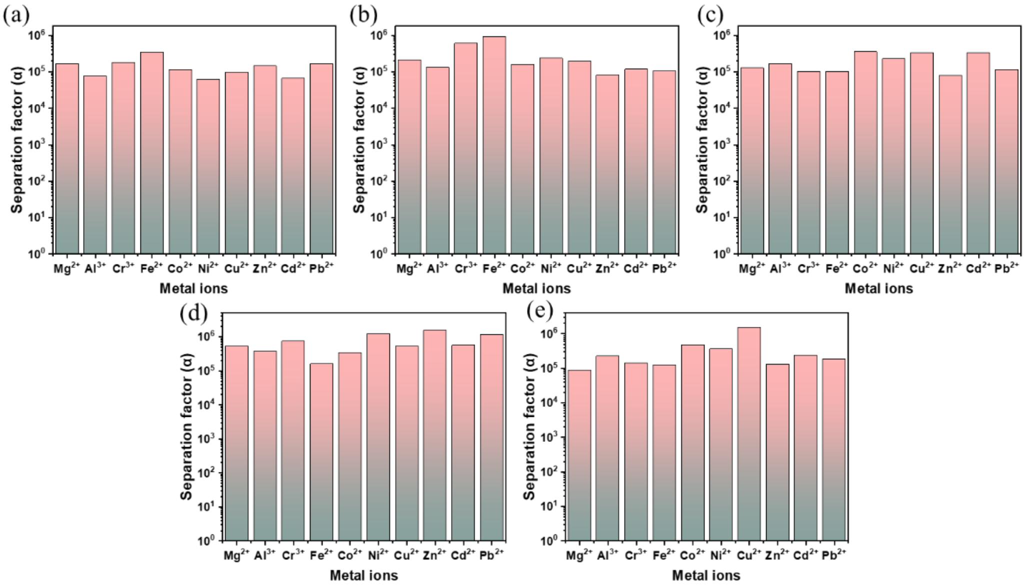

Figure S41. Adsorption separation coefficients (α) of **NAS–HBA** for different metals: (a) $NaAuCl_4$, (b) $KAuCl_4$, (c) NH_4AuCl_4, (d) $NaAuBr_4$ and (e) $KAuBr_4$.

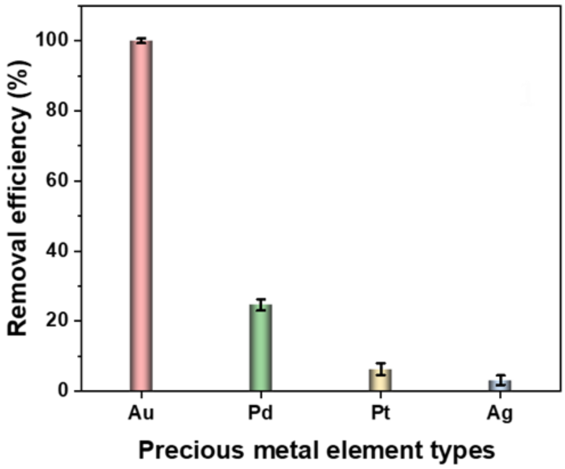

Figure S42. The adsorption efficiency of precious metal for **NAS－HBA** in pH=1 of aqueous solution.

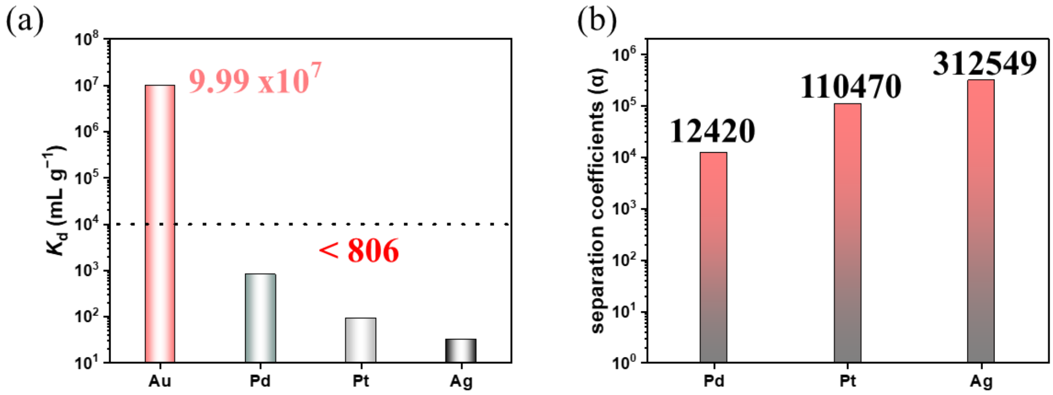

Figure S43. The distribution coefficients (K_d)(a) and adsorption separation coefficients (α) (b)of **NAS–HBA** for different noble metal.

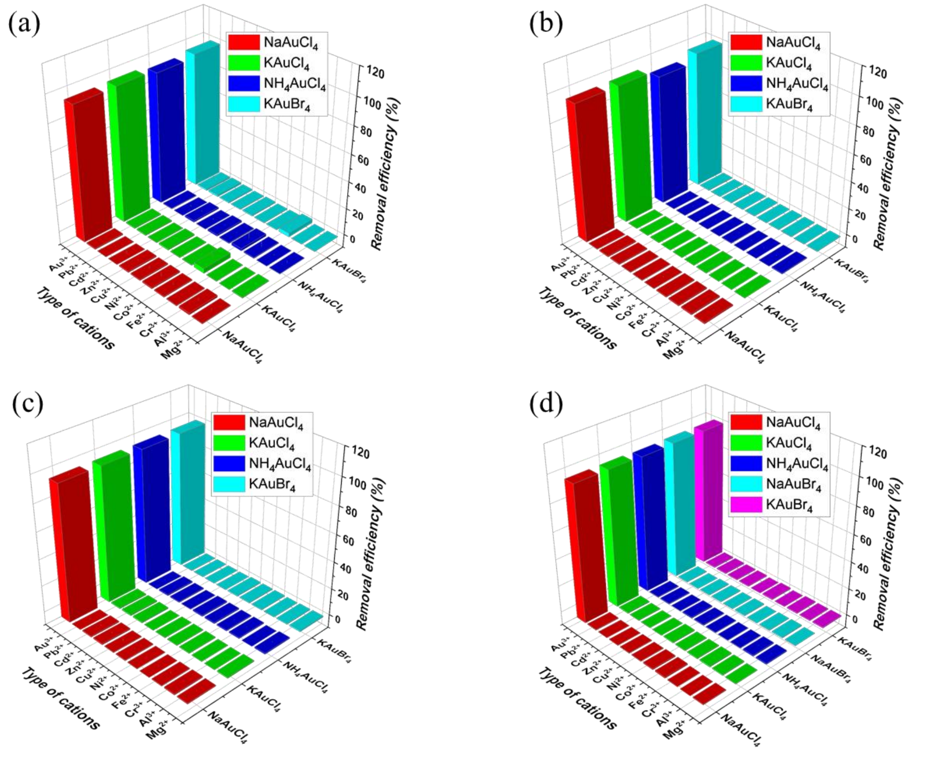

Figure S44. Adsorption of four types of Au(III) aqueous solutions containing different equivalents of competing cations (Mg^{2+}, Al^{3+}, Cr^{3+}, Fe^{3+}, Co^{2+}, Ni^{2+}, Cu^{2+}, Zn^{2+}, Cd^{2+} and Pb^{2+}) with **NAS–HBA**: (a) 10–fold, (b) 100–fold, (c) 200–fold and (d) 200–fold with 2 M HCl. The concentration of **NAS–HBA** was 1 mg/mL.

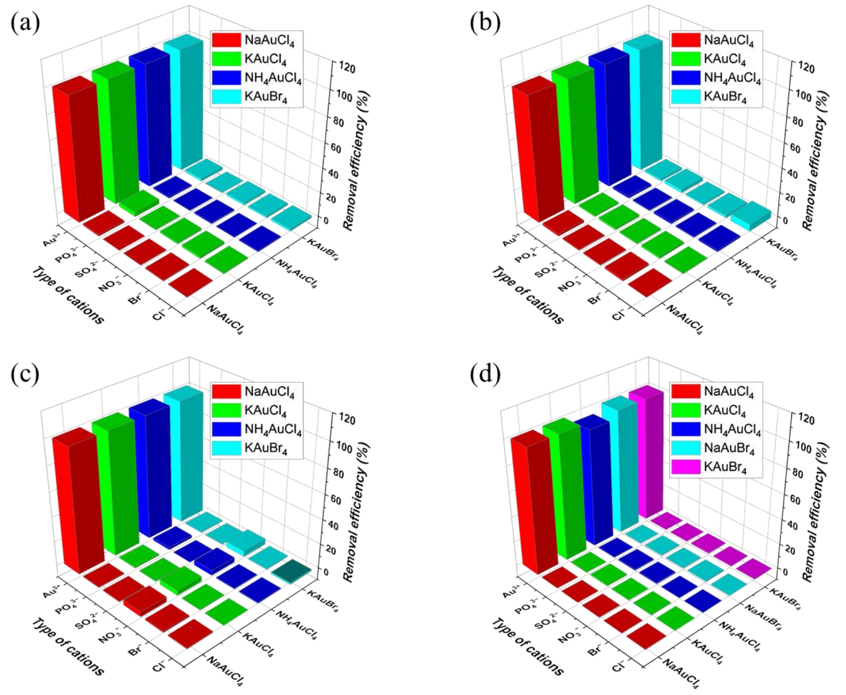

Figure S45. Adsorption of four types of Au(III) aqueous solutions containing different equivalents of competing anions (Cl^-, Br^-, NO_3^- and SO_4^{2-}) with **NAS–HBA**: (a) 10–fold, (b) 100–fold, (c) 200–fold and (d) 200–fold with 2 M HCl. The concentration of **NAS–HBA** was 1 mg/mL. Error bars represent SD; n = 3 independent experiments.

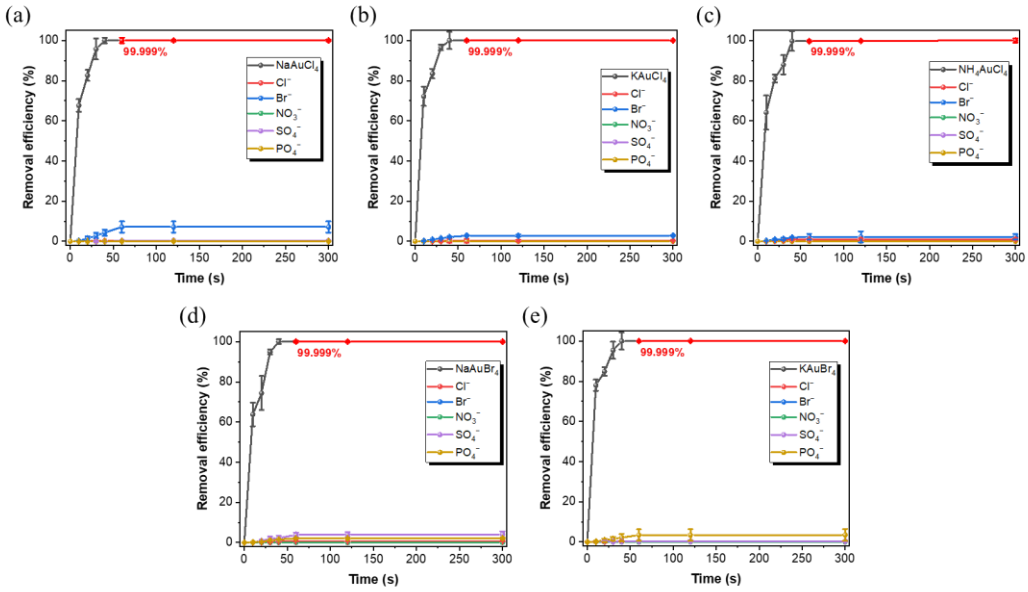

Figure S46. Adsorption of different types of Au(III) aqueous solutions (20 ppm) containing 1 equivalent of equal–molar competing anions (Cl^-, Br^-, NO_3^- and SO_4^{2-}) with **NAS–HBA** at pH 1: (a) $NaAuCl_4$, (b) $KAuCl_4$, (c) NH_4AuCl_4, (d) $NaAuBr_4$ and (e) $KAuBr_4$. The concentration of **NAS–HBA** was 1 mg/mL. Error bars represent SD; n = 3 independent experiments.

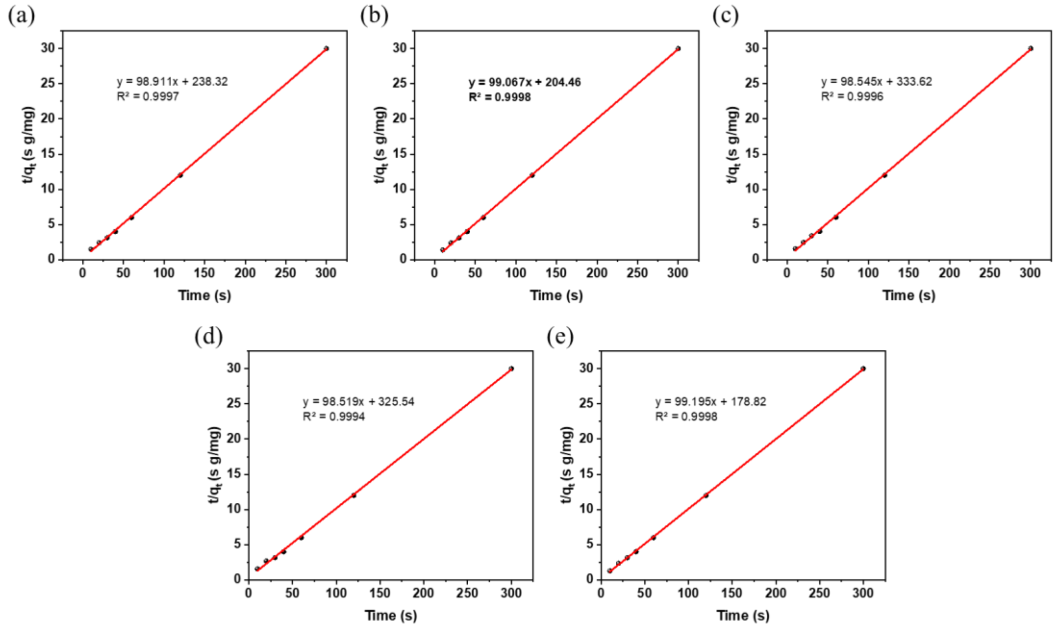

Figure S47. Pseudo–second order kinetic model of Au (III) adsorption from aqueous solutions with **NAS–HBA** at pH=1, (a) $NaAuCl_4$, (b) $KAuCl_4$, (c) NH_4AuCl_4, (d) $NaAuBr_4$ and (e) $KAuBr_4$. These data were derived from figure S46.

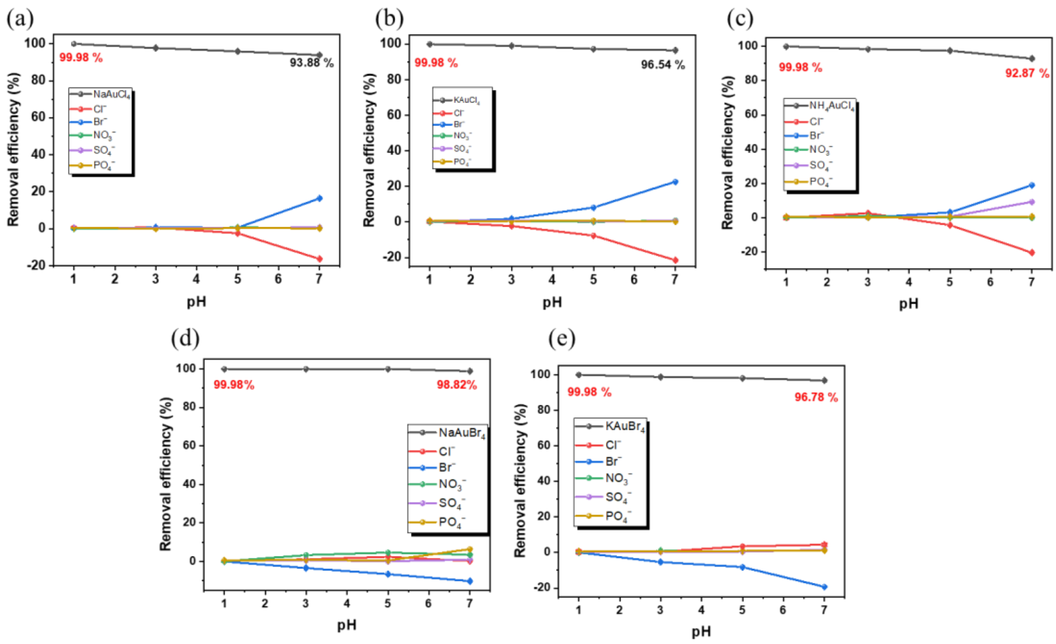

Figure S48. Adsorption of different types of Au(III) aqueous solutions (20 ppm) containing 1 equivalent of equal–molar competing anions (Cl^-, Br^-, NO_3^-, and SO_4^{2-}) with **NAS–HBA** at pH levels of 1, 3, 5, and 7: (a) $NaAuCl_4$, (b) $KAuCl_4$, (c) NH_4AuCl_4, (d) $NaAuBr_4$ and (e) $KAuBr_4$. The concentration of **NAS–HBA** was 1 mg/mL. Error bars represent SD; n = 3 independent experiments.

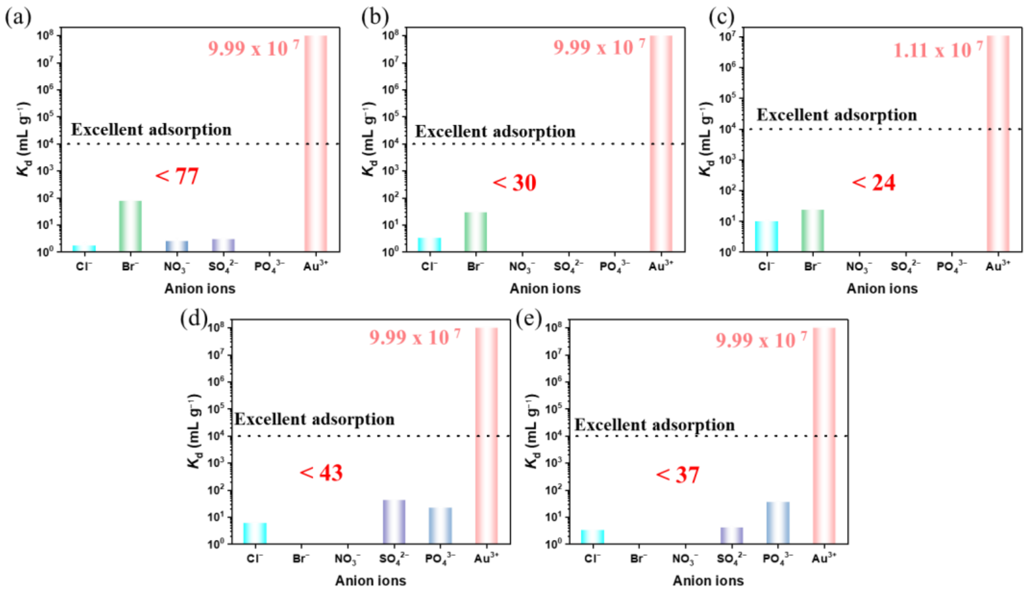

Figure S49. The distribution coefficients (K_d) of **NAS – HBA** for different anions adsorption efficiency: (a) $NaAuCl_4$, (b) $KAuCl_4$, (c) NH_4AuCl_4, (d) $NaAuBr_4$ and (e) $KAuBr_4$.

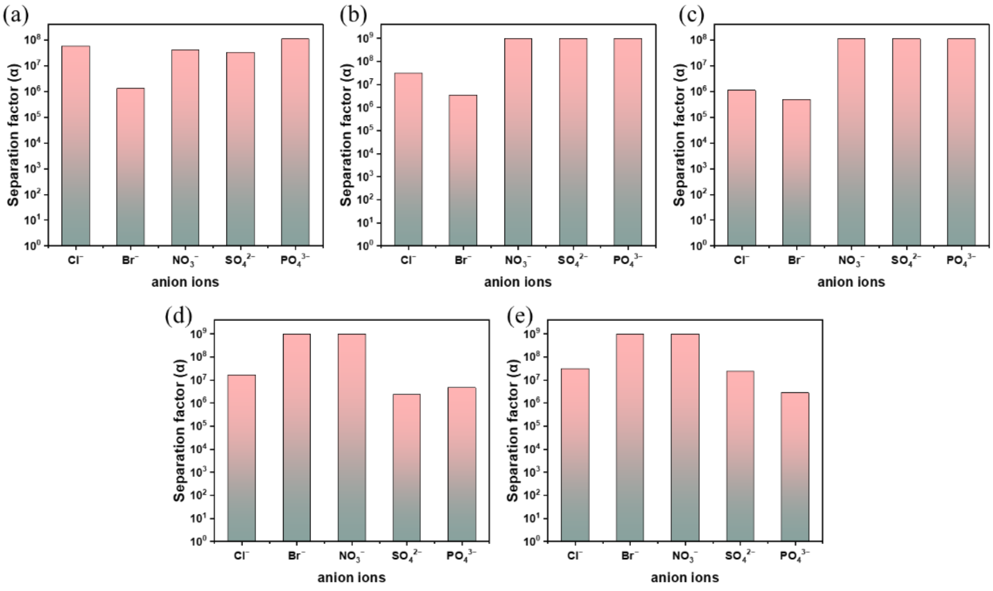

Figure S50. Adsorption separation coefficients (α) of **NAS–HBA** for different anions: (a) $NaAuCl_4$, (b) $KAuCl_4$, (c) NH_4AuCl_4, (d) $NaAuBr_4$ and (e) $KAuBr_4$.

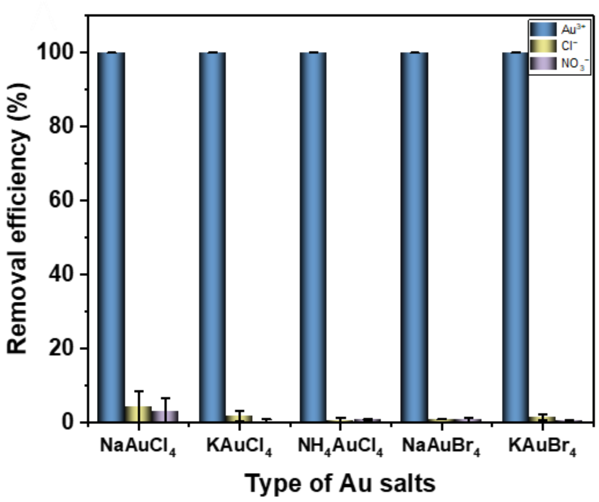

Figure S51. Adsorption of five types of Au(III) aqueous solutions ($NaAuCl_4$, $KAuCl_4$, NH_4AuCl_4, $NaAuBr_4$ or $KAuBr_4$) containing 2000 equivalents of competing anions (Cl^- and NO_3^-) with **NAS–HBA** at pH 1. The concentration of **NAS–HBA** was 1 mg/mL. Error bars represent SD; n = 3 independent experiments.

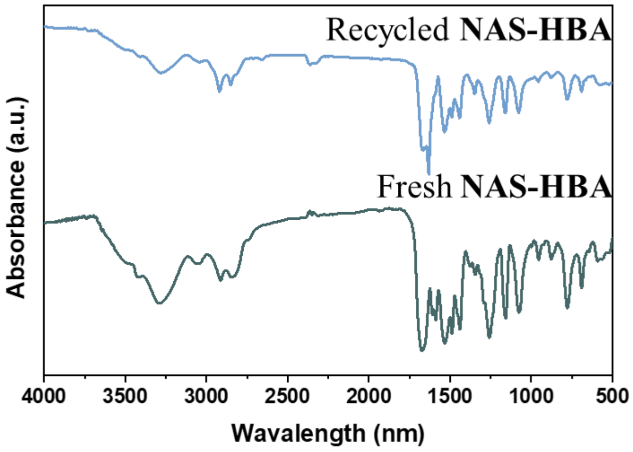

Figure S52. The FT－IR spectrum of fresh (bottom) and recycled (top) **NAS–HBA** after undergoing 30 adsorption–desorption cycles.

Table S3. Elemental composition and concentration in the actual system.

Types (ppm)	AMD CPU	PCB	Catalytic wastewater	Gold ores	AMD CPU (Py－NBS)
**Mg**	158.03	170.02	–	338.31	0.07
**Cu**	2920.07	11162.88	102.31	935.27	106
**Al**	195.80	212.40	31.94	865.73	0.06
**Si**	35.49	19.07	–	29.86	–
**Ca**	51.26	53.75	–	24.95	–
**Ti**	0.05	–	5.21	–	–
**Cr**	6.33	53.75	–	12.32	0.01
**Mn**	3.48	166.87	10.34	58.93	0.03
**V**	–	–	–	–	–
**Fe**	408.01	386.16	–	14065.93	0.71
**Co**	0.43	3.33	25.74	15.37	–
**Ni**	157.99	861.97	–	18.80	1.38

**Zn**	218.44	371.39	–	2793.19	–
**As**	–	–	–	–	–
**Sr**	0.07	29.61	–	0.06	–
**Y**	0.01	0.60	–	0.58	–
**Zr**	0.13	29.61	–	–	–
**Mo**	0.78	–	–	–	–
**Rh**	–	0.07	–	0.05	–
**Pd**	–	1.09	–	0.05	–
**Cd**	1.37	1.36	–	22.36	–
**In**	0.03	0.15	–	1.17	–
**Sb**	0.97	0.38	–	2.82	–
**Sn**	5.17	0.12	–	1.08	0.14
**Cs**	–	–	–	–	–
**Ba**	0.65	42.05	2.36	0.38	–
**W**	–	–	–	–	–
**Tl**	–	–	–	–	–
**Pb**	19.83	42.05	–	52.41	1.08
**Bi**	0.03	5.24	–	51.03	–
**Ag**	1.71	237.19	56.32	52.43	–
**Au**	1.28	2.52	121.41	1.58	1.8

– : not detected.

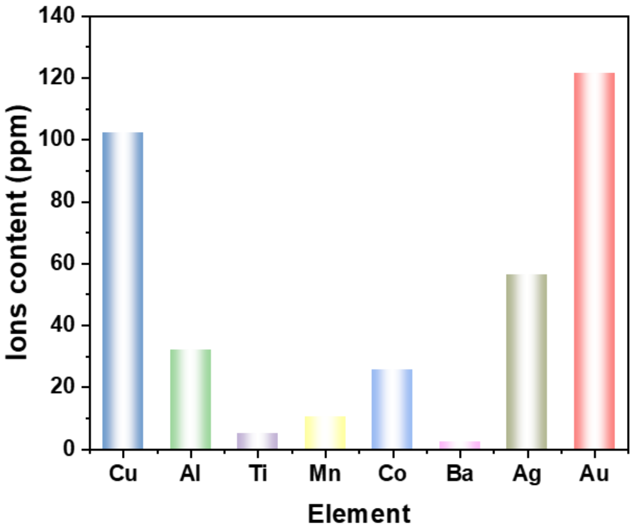

Figure S53. The actual content of each element in the catalytic wastewater.

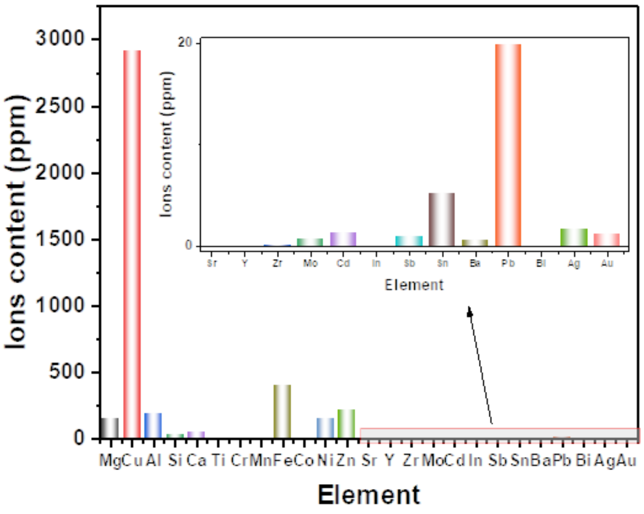

Figure S54. Actual content of each element in the AMD CPU leachates.

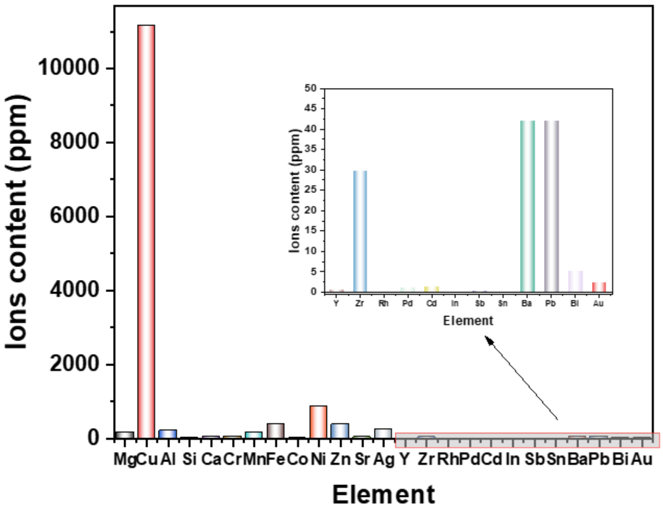

Figure S55. Actual content of each element in the PCB leachates.

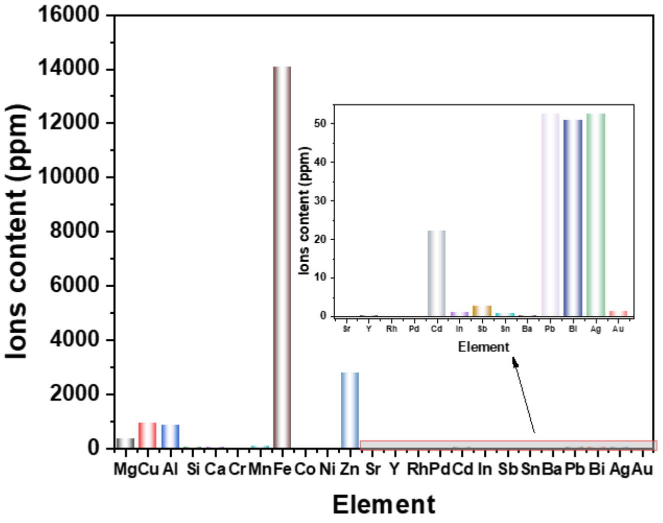

Figure S56. Actual content of each element in the gold ores leachates.

Table S4. Elemental composition and concentration in catalytic waste and PCB leachates.

elements	CW –*aqua regia* (ppm)	PCB–*aqua regia* (ppm)	PCB–Py–NBS (ppm)
**Mg**	1.50	1.65	1.81
**Al**	3.47	19.83	1.66
**Si**	8.61	20.12	–
**Ca**	10.69	67.68	–
**Sc**	7.33	8.78	–
**Ti**	0.69	15.51	–
**V**	–	8.56	–

**Cr**	0.12	110.46	0.05
**Mn**	0.12	5.83	0.66
**Fe**	23.86	28.01	17.63
**Co**	0.13	0.14	–
**Ni**	–	15.98	34.41
**Cu**	1.15	389.40	2649.51
**Zn**	–	5.83	5.43
**Sr**	–	22.91	–
**Y**	–	3.37	–
**Zr**	1.88	1.48	–
**Mo**	128.94	1.36	–
**Rh**	–	0.84	–
**Pd**	–	3.80	–
**Ag**	2.55	101.64	1.27
**In**	–	18.81	–
**Sn**	–	4557.30	3.59
**Sb**	–	12.15	–
**Ba**	–	110.92	–
**W**	–	1.15	–
**Pb**	–	8.08	27.15
**Bi**	–	9.55	–
**Au**	210.98	123.58	45.03

– : not detected.

5. X–ray experimental details

X–ray experimental for complex NAS–HBA – $AuBr_2^-$

Single crystals of complex $Br^-\cdot 2H_2O$**@NAS–HBA**$\cdot 2H^+\cdot AuBr_2^-$ were obtained as colorless plates via the liquid–solid extraction of $NaAuBr_4$ by a $CHCl_3$ solution of the receptor **NAS–HBA**. A suitable crystal was selected and the data were collected on a Bruker D8 VENTURE PHOTON 100 CMOS system equipped with a mirror monochromator and a Cu–Kα INCOATEC IμS micro focus source (λ = 1.54178 Å). The crystal was kept at 170 K during data collection. Using Olex2,[36] the structure was solved with the ShelXT[37] structure solution program using direct methods and refined with the ShelXL[38] refinement package using least squares minimization. Tables of positional and thermal parameters, bond lengths and angles, torsion angles and figures are in the CIF file. CCDC deposition number: 2321380.

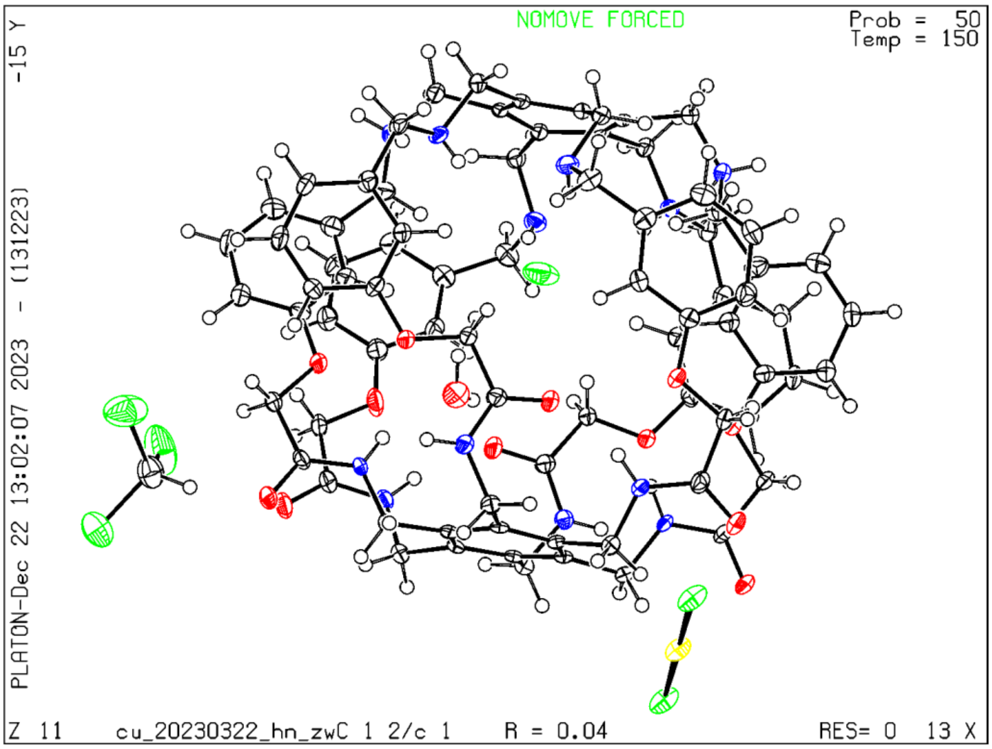

Figure S57. View of the complex $Br^-\cdot 2H_2O$**@NAS–HBA**$\cdot 2H^+\cdot AuBr_2^-$. Displacement ellipsoids are scaled to the 50% probability level

Table S5 Crystal data and structure refinement for $Br^{-}·2H_2O$**@NAS–HBA**$·2H^{+}·AuBr_2^{-}$

Identification code	$Br^{-}·2H_2O$**@NAS–HBA**$·2H^{+}·AuBr_2^{-}$
Empirical formula	$C_{80}H_{90}AuBr_3Cl_6N_{12}O_{14}$
Formula weight	1046.52
Temperature/K	150.0
Crystal system	monoclinic
Space group	C2/c
a/Å	21.5295(7)
b/Å	21.2877(7)
c/Å	22.3599(8)
α/°	90
β/°	93.830(2)
γ/°	90
Volume/Å^3	10225.0(6)
Z	4
ρcalcg/cm^3	1.360
μ/mm^{-1}	5.998
F(000)	4208.0
Crystal size/mm^3	0.23 × 0.21 × 0.17
Radiation	CuKα (λ = 1.54178 Å)
2Θ range for data collection/°	7.214 to 144.824
Index ranges	–26 ≤ h ≤ 21, –20 ≤ k ≤ 26, –27 ≤ l ≤ 27
Reflections collected	33431
Independent reflections	9965 [Rint = 0.0604, Rsigma = 0.0561]
Data/restraints/parameters	9965/7/539
Goodness–of–fit on F^2	1.061
Final R indexes [I>=2σ (I)]	R1 = 0.0416, wR2 = 0.1134
Final R indexes [all data]	R1 = 0.0490, wR2 = 0.1181
Largest diff. peak/hole / e Å^{-3}	0.72/–0.97
CCDC number	2321380

6. References

[1] J. Luo, X. Luo, M. Xie, H.–Z. Li, H. Duan, H.–G. Zhou, R.–J. Wei, G.–H. Ning, D. Li, *Nat. Commun.* **2022**, *13*.

[2] L. Zhang, Q.–Q. Zheng, S.–J. Xiao, J.–Q. Chen, W. Jiang, W.–R. Cui, G.–P. Yang, R.–P. Liang, J.–D. Qiu, *Chem. Eng. J.* **2021**, *426*.

[3] B. Wang, Y. Ma, W. Xu, K. Tang, *J. Hazard. Mater.* **2023**, *451*, 131051.

[4] X.–J. Li, W.–R. Cui, W. Jiang, R.–H. Yan, R.–P. Liang, J.–D. Qiu, *Chem. Eng. J.* **2021**, *422*.

[5] T. Ma, R. Zhao, Z. Li, X. Jing, M. Faheem, J. Song, Y. Tian, X. Lv, Q. Shu, G. Zhu, *ACS Appl. Mater. Interfaces* **2020**, *12*, 30474–30482.

[6] J. Cao, Z. Xu, Y. Chen, S. Li, Y. Jiang, L. Bai, H. Yu, H. Li, Z. Bian, *Angew. Chem., Int. Ed. Engl.* **2023**, *62*, e202302202.

[7] B. Pangeni, H. Paudyal, K. Inoue, H. Kawakita, K. Ohto, S. Alam, *Cellulose* **2011**, *19*, 381–391.

[8] A. Li, N. Zheng, T. Yang, J. Xie, L. Li, K. Tang, C. Zhou, *RSC Adv.* **2021**, *11*, 29807–29815.

[9] M. Liu, D. Jiang, Y. Fu, G. Z. Chen, S. Bi, X. Ding, J. He, B.–H. Han, Q. Xu, G. Zeng, *Angew. Chem., Int. Ed. Engl.* **2023**.

[10] S. Zhong, Y. Wang, T. Bo, J. Lan, Z. Zhang, L. Sheng, J. Peng, L. Zhao, L. Yuan, M. Zhai, W. Shi, *Chem. Eng. J.* **2023**, *455*.

[11] Z. Qin, H. Deng, R. Huang, S. Tong, *Chem. Eng. J.* **2022**, *428*.

[12] Z. Zuhra, S. Ali, S. Ali, H. Xu, R. Wu, Y. Tang, *Chem. Eng. J.* **2022**, *431*.

[13] Y. Bai, J. Yang, Q. Shuai, L. Huang, *Colloids Surf., A* **2023**, *657*.

[14] J. Qiu, C. Xu, X. Xu, Y. Zhao, Y. Zhao, Y. Zhao, J. Wang, *Angew. Chem., Int. Ed. Engl.* **2023**, e202300459.

[15] Y. Hong, D. Thirion, S. Subramanian, M. Yoo, H. Choi, H. Y. Kim, J. F. Stoddart, C. T. Yavuz, *Proc. Natl. Acad. Sci. U.S.A.* **2020**, *117*, 16174–16180.

[16] T. H. Tianwei Xue, Li Peng, Olga A. Syzgantseva, Ruiqing Li, Chengbin Liu, Daniel T. Sun, Guangkuo Xu, Rongxing Qiu, Yanliang Wang, Shuliang Yang, Jun Li, Jian–Rong Li, Wendy L. Queen., *Sci. Adv.* **2023**, *9*, eadg4923.

[17] D. Mei, B. Yan, *Small* **2023**, e2304811.

[18] R. Ding, J. Liu, T. Wang, X. Zhang, *Chem. Eng. J.* **2022**, *449*.

[19] G. Lin, S. Wang, L. Zhang, T. Hu, J. Peng, S. Cheng, L. Fu, *J. Mol. Liq.* **2018**, *258*, 235–243.

[20] L. Yuan, N. Zheng, T. Yang, A. Li, Y. Yuan, J. Hua, L. Li, C. Zhou, *J. Taiwan Inst. Chem. Eng.* **2023**, *144*.

[21] H. L. Qian, F. L. Meng, C. X. Yang, X. P. Yan, *Angew. Chem., Int. Ed. Engl.* **2020**, *59*, 17607–17613.

[22] M. Can, M. Doğan, M. İmamoğlu, M. Arslan, *React. Funct. Polym.* **2016**, *109*, 151–161.

[23] L. Zhang, J.–Q. Fan, Q.–Q. Zheng, S.–J. Xiao, C.–R. Zhang, S.–M. Yi, X. Liu, W. Jiang, Q.–G. Tan, R.–P. Liang, J.–D. Qiu, *Chem. Eng. J.* **2023**, *454*.

[24] D. T. Sun, N. Gasilova, S. Yang, E. Oveisi, W. L. Queen, *J. Am. Chem. Soc.* **2018**, *140*, 16697–16703.

[25] H.–Y. Kong, Y. Tao, X. Ding, B.–H. Han, *Chem. Eng. J.* **2023**, *463*.

[26] A. M.E.H, X. Y. Mbianda, A. F. Mulaba–Bafubiandi, L. Marjanovic, *Hydrometallurgy* **2013**, *140*, 1–13.

[27] Y. Dai, K. Zheng, Y. Tan, W. Xiang, B. Xianyu, H. Xu, *Adv. Sustainable Syst.* **2020**, *4*.

[28] C. Wang, C. Xiong, X. Zhang, Y. He, J. Xu, Y. Zhao, S. Wang, J. Zheng, *Sep. Purif. Technol.* **2022**, *296*.

[29] X. Wu, H. Lin, F. Dai, R. Hu, B. Z. Tang, *CCS Chem.* **2020**, *2*, 191–202.

[30] Y. Chen, J. Tang, S. Wang, L. Zhang, *J. Mol. Liq.* **2022**, *349*.

[31] M. Mon, J. Ferrando–Soria, T. Grancha, F. R. Fortea–Pérez, J. Gascon, A. Leyva–Pérez, D. Armentano, E. Pardo, *J. Am. Chem. Soc.* **2016**, *138*, 7864–7867.

[32] Z. Zhou, W. Zhong, K. Cui, Z. Zhuang, L. Li, L. Li, J. Bi, Y. Yu, *Chem. Commun.* **2018**, *54*, 9977–9980.

[33] C. Wang, G. Lin, J. Zhao, S. Wang, L. Zhang, *Chem. Eng. J.* **2020**, *388*.

[34] H. Li, Y. Pan, F. Wu, Y. Zhou, J. Pan, *Green Chem.* **2022**, *24*, 7592–7601.

[35] N. F. Abd Razak, M. Shamsuddin, S. L. Lee, *Chem. Eng. Res. Des.* **2018**, *130*, 18–28.

[36] O. V. Dolomanov, L. J. Bourhis, R. J. Gildea, J. A. K. Howard, H. Puschmann, *J. Appl. Crystallogr.* **2009**, *42*, 339–341.

[37] G. M. Sheldrick, *Acta Crystallogr., Sect. A: Found. Adv.* **2015**, *71*, 3–8.

[38] G. M. Sheldrick, *Acta Crystallogr., Sect. C: Struct. Chem.* **2015**, *71*, 3–8.
